# An eco‐evolutionary optimality model explains the acclimated temperature response of photosynthesis

**DOI:** 10.1111/nph.71062

**Published:** 2026-04-02

**Authors:** Wenyao Gan, Nabil Alizadeh, Martin Best, Pier Luigi Vidale, I. Colin Prentice, Sandy P. Harrison

**Affiliations:** ^1^ Department of Geography and Environmental Science University of Reading Reading RG6 6AB UK; ^2^ Department of Life Sciences, Georgina Mace Centre for the Living Planet Imperial College London Silwood Park Campus, Buckhurst Road Ascot SL5 7PY UK; ^3^ Meteorological Office Fitzroy Road Exeter EX1 3PB UK; ^4^ Department of Meteorology University of Reading Reading RG6 6AB UK

**Keywords:** acclimated temperature response, eco‐evolutionary optimality, growth temperature, photosynthetic temperature responses, plant acclimation, *V*_cmax_, *J*_max_

## Abstract

- The optimal temperature of photosynthesis (*T*_opt_) generally increases with plant growth temperature. Changes in *T*_opt_ are associated with changes in the maximum carboxylation capacity at 25°C (*V*_cmax25_) and the maximum electron transport rate at 25°C (*J*_max25_). The ratio between *J*_max25_ and *V*_cmax25_ declines with warming. Accurate representation of leaf‐level photosynthetic responses to temperature is essential for realistic projections of the terrestrial carbon cycle and its response to ongoing climate changes. However, many land surface models incorporate thermal acclimation through empirical approaches and through assigning distinct but static parameter values to plant functional types (PFTs). Eco‐evolutionary optimality (EEO) approaches provide a simpler way of modelling photosynthesis without recourse to PFTs.
- Here, we use the subdaily P model, an EEO‐based model of photosynthesis that explicitly separates the instantaneous and acclimated responses of photosynthetic parameters to temperature to investigate how optimal temperature changes with growth temperature, as represented by leaf or air temperature.
- We show that the simulated responses are consistent with observations from both controlled experiments and eddy covariance flux tower data.
- We show that changes in *T*_opt_, and in the assimilation rate at *T*_opt_, are caused by changes in carboxylation capacity and electron transport rate that follow directly from the hypotheses underlying the model.

## Introduction

Terrestrial ecosystems are currently a major global carbon sink (Friedlingstein *et al*., 2024), but projections of their future role are highly uncertain due in part to poorly constrained photosynthetic responses to warming (Lombardozzi *et al*., 2015; Arora *et al*., 2020; McDowell *et al*., 2020). Leaf‐level photosynthetic temperature responses are among the most important physiological processes driving carbon uptake in these models (Rogers *et al*., 2017; Smith & Dukes, 2017). In particular, the optimal temperature of photosynthesis (*T*_opt_) and the maximum rate of photosynthesis (*A*_opt_) will strongly influence the response of gross primary production (GPP) to warming (Rogers *et al*., 2017; Smith & Dukes, 2017).

Many observational and experimental studies have shown that the *T*_opt_ increases with increasing plant growth temperature. This shift is achieved through acclimation, a reversible physiological adjustment that enables plants to maintain function under changing thermal environments. Thermal acclimation of photosynthesis has been documented across a wide range of ecosystems (Drake *et al*., 2015, 2017; Kroner & Way, 2016; Slot & Winter, 2017; Benomar *et al*., 2018; Kurepin *et al*., 2018; Reich *et al*., 2018; Dusenge *et al*., 2020; Carter *et al*., 2021). Shifts in *T*_opt_ have been associated with changes in the ratio of maximum electron transport rate at 25°C (*J*_max25_) to maximum carboxylation capacity at 25°C (*V*_cmax25_). This ratio declines with increasing growth temperature (Kattge & Knorr, 2007; Wang *et al*., 2017; Crous *et al*., 2022). Other thermodynamic parameters including the activation energies (*E*_a_), rates of deactivation (*H*_d_) and entropy term (Δ*S*) for *V*_cmax_ and *J*_max_ have also been found to adjust in response to growth temperature (Kattge & Knorr, 2007; Kumarathunge *et al*., 2019; Crous *et al*., 2022).

Many land surface models (LSMs) implement these temperature responses via static parameters assigned by plant functional type (PFT), without accounting for the full range of plant responses across timescales from instantaneous biochemical adjustments to short‐term acclimation and long‐term evolutionary adaptation. This simplification can result in model responses that do not reflect observed plant plasticity, particularly with respect to changes in the maximum rate of *A*_opt_. LSMs that incorporate thermal acclimation rely on empirical approaches, specifically the method proposed by Kattge & Knorr (2007) (Lombardozzi *et al*., 2015; Smith *et al*., 2016; Mercado *et al*., 2018) and subsequently updated by Kumarathunge *et al*. (2019) (Bennett *et al*., 2024). These models simulate acclimation by modifying the temperature response functions of *V*_cmax_ and *J*_max_, resulting in shifts in *T*_opt_. Kumarathunge *et al*. (2019) included a larger number of PFTs than previous approaches and distinguished between acclimation to recent growth temperature and longer term adaptation to the temperature of origin. While these empirical relationships improve model flexibility compared with fixed‐parameter approaches (Smith & Keenan, 2020), they do not offer a unified explanation for why plants adjust their thermal responses.

Eco‐evolutionary optimality (EEO) theory provides a simpler basis for modelling plant responses to environmental changes, without the need to rely on empirical relationships or consideration of the underlying processes (Harrison *et al*., 2021). EEO theory assumes that plants adjust their traits to maximise competitive fitness under specific conditions. The measure chosen to characterise optimal performance varies between EEO models: Some models focus on optimising photosynthesis (Medlyn, 1996), some net primary production (Franklin, 2007; Potkay & Feng, 2023), some net carbon gain (Prentice *et al*., 2014; Smith & Dukes, 2017; Sperry *et al*., 2017; Venturas *et al*., 2018) and some water‐use (Sperry *et al*., 2017) or water transport efficiencies (Koçillari *et al*., 2021). Nevertheless, all of these approaches enable plant physiological traits to emerge from environmental conditions rather than being prescribed.

The P model (Prentice *et al*., 2014; Wang *et al*., 2017; Stocker *et al*., 2020) is an EEO‐based model of GPP, which has been shown to capture broad‐scale spatial and seasonal patterns in photosynthetic traits despite the fact that it only distinguishes two PFTs based on their photosynthetic pathway, namely C_3_ and C_4_ plants (Smith *et al*., 2019; Dong *et al*., 2023; Cai *et al*., 2025). A subdaily implementation of the P model (Mengoli *et al*., 2022), run on a half‐hourly time step for instantaneous responses and in which plants acclimate to past conditions on timescales of *c*. 15 d, has been coupled in the Noah land surface model with multiparameterization options (NOAH‐MP LSM) (Ren *et al*., 2025). Comparison of the two schemes at half‐hourly, monthly and annual timescales across all the FLUXNET2015 sites showed that the EEO‐scheme performed better than the default NOAH‐MP scheme overall. The standard NOAH‐MP scheme underestimates monthly GPP across the FLUXNET2015 sites by 10%, and this was reduced to < 2% in the EEO‐based implementation. Similarly, on an annual timestep, the agreement with observations improved from an *R* ^2^ of 0.57 in the standard model to 0.66 in the EEO‐based implementation. Ren *et al*. (2025) also showed that the EEO‐based implementation explained 62% of the temporal and 70% of the spatial variation in *V*_cmax25_.

In this study, we used the subdaily P model run on a half‐hourly or hourly timestep to investigate the optimal behaviour of the photosynthetic temperature response. We first simulated temperature–response curves under idealised, controlled conditions where only growth temperature was varied, and all other factors were held constant. We then examined how the *T*_opt_, *A*_opt_ and the ratio of *J*_max25_ and to *V*_cmax25 _and change with growth temperature, comparing these patterns with leaf‐level gas exchange measurements. We also evaluated the model predictions against ecosystem‐scale flux data to assess how well the model captures temperature acclimation at larger scales. The P model was run using leaf temperature for the gas exchange comparison and *T*_air_ for the flux tower data comparisons. This then allows us to diagnose how the change in optimal growth temperature is related to changes in *V*_cmax_ and *J*_max_.

## Materials and Methods

We conducted two sets of analyses to investigate the mechanisms of photosynthetic temperature acclimation. The first set of simulations in which all factors except growth temperature were held constant provides a theoretical baseline for understanding how photosynthetic traits acclimate to temperature. Second, we compared the model outputs with two independent datasets: leaf‐level gas exchange measurements from controlled chamber experiments and ecosystem‐scale flux data. This approach allows us to compare model predictions with observations and assess how temperature acclimation is represented in both the model and observations.

### The subdaily P model

**Fig. 1:**
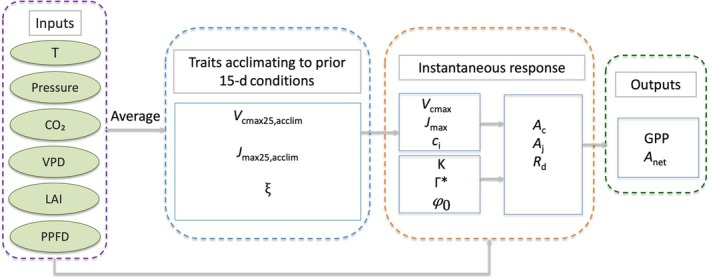
Workflow of subdaily P model framework. The inputs are air/leaf temperature (*T*), air pressure (Pressure), atmospheric CO_2_ concentration (CO_2_), vapour pressure deficit (VPD), the Leaf Area Index (LAI), and available light (photosynthetic photon flux density (PPFD)). The outputs are 30‐min gross primary production (GPP) and net photosynthesis (*A*_net_). Three traits are acclimating to the past 15‐d midday condition: *V*_cmax25,acclim_, *J*_max25,acclim_, and the optimal stomatal parameter ξ.

The subdaily P model (Mengoli *et al*., 2022) builds on the P model framework (Prentice *et al*., 2014; Wang *et al*., 2017; Stocker *et al*., 2020) by explicitly separating the instantaneous and acclimated responses of photosynthetic responses to environmental variation (Fig. 1). Acclimated variables include acclimated *V*_cmax25_ (*V*_cmax25,acclim_) and *J*_max25_ (*J*_max25,acclim_) and the optimal stomatal parameter ξ. Eqns (Eqn 1), (Eqn 2), (Eqn 3), (Eqn 4), (Eqn 5), (Eqn 6), (Eqn 7), (Eqn 8), (Eqn 9), (Eqn 10), (Eqn 11), (Eqn 12) describe the instantaneous response of photosynthesis. To simulate the instantaneous response, photosynthesis is calculated as the minimum of the Rubisco‐limited rate and the light‐limited rate, following the standard Farquhar–von Caemmerer–Berry model of C_3_ photosynthesis (FvCB: Farquhar *et al*., 1980). The Rubisco‐limited rate, representing the rate of CO_2_ fixation determined by the availability and catalytic activity of Rubisco enzyme, is given by:

(Eqn 1) $$A_{c}=V_{\text{cmax}}\times \frac{\left(c_{i}-\Gamma^{*}\right)}{\left(c_{i}+K\right)}$$

and the light‐limited rate by:

(Eqn 2) $$A_{J}=\frac{J}{4}\times \frac{\left(c_{i}-\Gamma^{*}\right)}{\left(c_{i}+2\Gamma^{*}\right)}$$

where *V*_cmax_ represents the maximum rate of RuBisCO carboxylation and *c*_i_ is the leaf‐internal CO_2_ partial pressure. Here, instantaneous *c*_i_ is mainly determined by two variables, the current vapour pressure deficit (VPD) and the acclimated optimal stomatal parameter ξ (Eqn 12). ξ is kept constant over the diurnal cycle, since this is a trait that adjusts on acclimation timescales rather than responding instantaneously to short‐term fluctuations in environmental drivers. $\Gamma^{*}$ is the photorespiratory compensation point, and *K* is the effective Michaelis–Menten coefficient of RuBisCO. The electron transport rate *J* is calculated as:

(Eqn 3) $$J=\frac{4\phi_{0}I_{\mathrm{abs}}}{\sqrt{1+{\left(\frac{4\phi_{0}I_{\mathrm{abs}}}{J_{\max}}\right)}^{2}}}$$

(Eqn 4) $$\phi_{0}\left(T\right)=\frac{1}{8}\times \left(0.352+0.022\cdot T-0.00034\cdot T^{2}\right)$$

where $\phi_{0}$ is the intrinsic quantum efficiency, *I*_abs_ is absorbed PAR, and *J*_max_ is the maximum electron transport capacity.

Temperature sensitivities for Γ* and *K* are taken from Farquhar *et al*. (1980) and Bernacchi *et al*. (2001). $\Gamma^{*}\left(T\right)$ is calculated from the CO_2_ compensation point at 25°C ($\Gamma_{25}^{*}$) as

(Eqn 5) $$\Gamma^{*}\left(T\right)=\Gamma_{25}^{*}\cdot \exp\frac{\left(T-25\right)\cdot E_{a,\Gamma^{*}}}{R\cdot 298\left(T+273.15\right)}\cdot \frac{\text{Pres}}{\mathrm{Pa}\left(O\right)}$$

where $\Gamma_{25}^{*}$= 4.322 Pa, $E_{a,\Gamma^{*}}$ = 37 830 J mol^−1^ is the activation energy for Γ*, *R* = 8.314 J mol^−1^ K^−1^ is the universal gas constant (Farquhar *et al*., 1980). Pres is surface air pressure and Pa(O) is the partial pressure of O_2_.

*K* is calculated from the Michaelis–Menten constants for carboxylation (*K*_c_) and oxygenation (*K*_o_) using Eqn 6:

(Eqn 6) $$K=K_{c}\left(1+\frac{\mathrm{Pa}\left(O\right)\;}{K_{o}}\right)$$

The temperature dependence of *K*_c_ and *K*_o_ follows:

(Eqn 7) $$K_{c}\left(T\right)=K_{c,25}\cdot \exp\frac{\left(T-25\right)\cdot E_{a,\mathrm{kc}}}{R\cdot 298\left(T+273.15\right)}$$

(Eqn 8) $$K_{o}\left(T\right)=K_{o,25}\cdot \exp\frac{\left(T-25\right)\cdot E_{a,\mathrm{ko}}}{R\cdot 298\left(T+273.15\right)}$$

where *K*_c,25_ is the Michaelis–Menten constant for Rubisco carboxylation at 25°C and *K*_o,25_ at 25°C is the Michaelis–Menten constant for Rubisco oxygenation at 25°C. *K*_c,25_ and *K*_o,25_ is set to 39.97 Pa and 27 480 Pa, respectively. *E*_a,kc_ and *E*_a,ko_ are the activation energies for *K*_c_ and *K*_o_, set to 79 430 and 36 380 J mol^−1^, respectively.

Net photosynthesis rate is calculated as:

(Eqn 9) $$A_{\mathrm{net}}=A-R_{d}$$

where leaf dark respiration (*R*_d_) is estimated as:

(Eqn 10) $$R_{d}=0.015\times V_{\text{cmax}}$$

The instantaneous values of *V*_cmax_ and *J*_max_ are adjusted for the current leaf temperature (*T*_ins_) using basic Arrhenius equations:

(Eqn 11) $$V_{\text{cmax}}\left(T_{\mathrm{ins}}\right)=V_{\text{cmax}25,\text{acclim}}\;\times \exp\left(\frac{E_{a,v}}{R}\times \frac{T_{\mathrm{ins}}-298.15}{T_{\mathrm{ins}}\times 298.15}\right)$$

(Eqn 12) $$J_{\max}\left(T_{\mathrm{ins}}\right)=J_{\max25,\text{acclim}}\;\times \exp\left(\frac{E_{a,J}}{R}\times \frac{T_{\mathrm{ins}}-298.15}{T_{\mathrm{ins}}\times 298.15}\right)$$

where *E*_a,v_ = 65 330 J ${\mathrm{mol}}^{-1}$ is the activation energy for *V*_cmax_ (Bernacchi *et al*., 2001), and *E*_a,J_ = 43 900 J ${\mathrm{mol}}^{-1}$ is the activation energy for *J*_max_ (Bernacchi *et al*., 2003). Results using the basic form of the Arrhenius equation do not differ significantly from those that would be obtained using the more complex peaked formulation over the range of growth temperatures considered here (Ren *et al*., 2024).

The *V*_cmax25,acclim_, and *J*_max25,acclim_ values were calculated by applying the inverse form of the Arrhenius equation to *V*_cmax,opt_ and *J*_max,opt_ at the acclimated temperature (*T*_accl_):

(Eqn 13) $$V_{\text{cmax}25,\text{acclim}}\left(T_{\text{accl}}\right)==V_{\text{cmax},\mathrm{opt}}\times \exp\left(\frac{E_{a,v}}{R}\times \frac{298.15-T_{\text{accl}}\;}{T_{\text{accl}}\times 298.15}\right)$$

(Eqn 14) $$J_{\max25,\text{acclim}}\left(T_{\text{accl}}\right)=J_{\max,\mathrm{opt}}\times \exp\left(\frac{E_{a,J}}{R}\times \frac{298.15-T_{\text{accl}}\;}{T_{\text{accl}}\times 298.15}\right)$$

Here, *V*_cmax,opt_ and *J*_max,opt_ represent the *V*_cmax_ and *J*_max_ at the average midday (11:30–12:30 local solar time, corresponding to the period of peak irradiance) environmental conditions during the previous 15 d as well as the *T*_accl_.

*V*_cmax,opt_ and *J*_max,opt_ are determined using two complementary optimality principles. According to the coordination hypothesis, plants adjust their photosynthetic capacities so that *A*_J_ is approximately equal to *A*_c_, ensuring efficient use of available light (Haxeltine & Prentice, 1996; Maire *et al*., 2012). Interpretation of the coordination hypothesis as an optimality can be found in Notes S1. The least‐cost hypothesis, as formulated in Prentice *et al*. (2014), predicts that the *c*_i_ adjusts in response to ambient CO_2_ partial pressure (*c*_a_), VPD, temperature and atmospheric pressure, thereby determining the optimal stomatal sensitivity (ξ) that balances carbon assimilation and water loss (Eqn 15). A higher ξ reduces the sensitivity of *c*_i_ to changes in VPD. This balance minimises the combined costs of carboxylation and water transport required to sustain a given assimilation rate (Prentice *et al*., 2014):

(Eqn 15) $$\xi =\sqrt{\frac{\beta \left(K+\Gamma^{*}\;\right)}{1.6\eta^{*}}}$$

β is the unitless cost ratio for maintaining carboxylation and water transport (here taken to be 146, see Wang *et al*., 2017) at 25°C. η* is the unitless ratio of the viscosity of water at a given temperature relative to its viscosity at 25°C (Huber *et al*., 2009).

Based on these principles, we can derive *V*_cmax,opt_ and *J*_max,opt_ and *c*_i,opt_, where the ‘opt’ subscript denotes variables defined at the growth temperature and all temperature‐dependent terms are evaluated accordingly (Eqns (Eqn 16), (Eqn 17), (Eqn 18)):

(Eqn 16) $$V_{\text{cmax},\mathrm{opt}}=\frac{\phi_{0}I_{\mathrm{abs}}[\left(c_{i,\mathrm{opt}}+K\right)}{\left(c_{i,\mathrm{opt}}+2\Gamma^{*}\right)}\;\sqrt{\left\{1-{\left[c^{*}\frac{\left(c_{i,\mathrm{opt}}+2\Gamma^{*}\right)}{\left(c_{i,\mathrm{opt}}-\Gamma^{*}\right)}\right]}^{\frac{2}{3}}\right\}}$$

(Eqn 17) $$J_{\max,\mathrm{opt}}=\frac{4\varphi_{0}I_{\mathrm{abs}}}{\sqrt{\frac{1}{\left\{1-{\left[c^{*}\frac{\left(c_{i,\mathrm{opt}}+2\Gamma^{*}\right)}{\left(c_{i,\mathrm{opt}}-\Gamma^{*}\right)}\right]}^{\frac{2}{3}}\right\}}}-1}$$

Here, *c*_i,opt_ is the leaf‐internal CO_2_ partial pressure corresponding to the optimal environmental conditions at which *V*_cmax,opt_, *J*_max,opt_ are determined.

(Eqn 18) $$c_{i,\mathrm{opt}}=\frac{\xi C_{a}+\Gamma^{*}\sqrt{\mathrm{VPD}}}{\xi +\sqrt{\mathrm{VPD}}}$$

where c* is a cost factor for electron transport capacity and has been estimated as 0.41 ± 0.112 from observed *J*_max_:*V*_max_ ratios (Wang *et al*., 2017). The instantaneous $c_{i}$ follows the same mathematical formulation as Eqn 18; it is calculated using instantaneous environmental forcing ($C_{a}$, $\Gamma^{*}$, and VPD) combined with the acclimated ξ.

The mathematical derivation of the optimal parameter ξ is described in Notes S2, and the derivations for optimal $V_{\text{cmax}}$ and $J_{\max}$ are detailed in Notes S3. Model parameters are described in Table 1. Model simulations were run in Python 3.10 using the Pyrealm code (Orme, 2024).

**Table 1:** Details of parameters, rates and constants used in the P model.

Symbol	Description	Unit or value in P model	References
*A*	Assimilation rate	μmol CO_2_ m^−2^ s^−1^	Farquhar *et al*. (1980)
*A*_C_	Carboxylation‐limited photosynthetic rate	μmol CO_2_ m^−2^ s^−1^	Farquhar *et al*. (1980)
*A*_J_	Electron transport‐limited photosynthetic rate	μmol CO_2_ m^−2^ s^−1^	Farquhar *et al*. (1980)
*A*_net_	Net photosynthesis rate	μmol CO_2_ m^−2^ s^−1^	Farquhar *et al*. (1980)
c*	Cost factor for electron transport capacity	0.41	Wang *et al*. (2017)
*c*_a_	Ambient CO_2_ partial pressure	Pa	
*c*_i_	Leaf‐internal CO_2_ partial pressure	Pa	Farquhar *et al*. (1980)
$E_{a,\Gamma^{*}}$	Activation energy for Γ*	37 830 J mol^−1^	Bernacchi *et al*. (2001)
$E_{a,j}$	Activation energy for *J*_max_	43 990 J mol^−1^	Bernacchi *et al*. (2003)
$E_{a,ko}$	Activation energy for *K*o	36 380 J mol^−1^	Bernacchi *et al*. (2001)
$E_{a,v}$	Activation energy for *V*_cmax_	65 330 J mol^−1^	Bernacchi *et al*., 2001
*J*	Rate of electron transport	μmol photon m^−2^ s^−1^	Farquhar *et al*. (1980)
*J*_max_	Maximum rate of electron transport	μmol photon m^−2^ s^−1^	Wang *et al*. (2017)
$J_{\max25}$	Maximum rate of electron transport at 25°C	μmol CO_2_ m^−2^ s^−1^	Wang *et al*. (2017)
$J_{\max25,\text{acclim}}$	Acclimated maximum rate of electron transport at 25°C representing photosynthetic capacity adjusted to previous 15‐d optimal environmental conditions	μmol CO_2_ m^−2^ s^−1^	
*K*	The effective Michaelis–Menten coefficient of Rubisco	Pa	Farquhar *et al*. (1980)
*K*_C_	Michaelis–Menten constant for carboxylation	Pa	Farquhar *et al*. (1980); Bernacchi *et al*. (2001)
*K*_C,25_	Michaelis–Menten constant for carboxylation at 25°C	39.97 Pa	Bernacchi *et al*. (2001)
*K*_O_	Michaelis–Menten constant for oxygenation	Pa	Farquhar *et al*. (1980); Bernacchi *et al*. (2001)
*K*_o,25_	Michaelis–Menten constant for oxygenation at 25°C	27 480 Pa	Bernacchi *et al*. (2001)
*R*	Universal gas constant	8.314 J mol^−1^ k^−1^	Farquhar *et al*. (1980)
*R*_d_	Respiration rate for a single leaf	μmol CO_2_ m^−2^ s^−1^	Farquhar *et al*. (1980)
*V*_cmax_	Maximum rate of carboxylation	μmol CO_2_ m^−2^ s^−1^	Farquhar *et al*. (1980)
$V_{\text{cmax}25}$	Maximum rate of carboxylation at 25°C	μmol CO_2_ m^−2^ s^−1^	Farquhar *et al*. (1980)
$V_{\text{cmax}25,\text{acclim}}$	Acclimated maximum rate of carboxylation at 25°C, representing photosynthetic capacity adjusted to previous 15‐d optimal environmental conditions	μmol CO_2_ m^−2^ s^−1^	
β	The ratio of the cost factors for carboxylation and transpiration capacities at 25°C	146	Stocker *et al*. (2020)
Γ*	Photorespiratory compensation point	Pa	Farquhar *et al*. (1980)
Γ*_25_	Photorespiratory compensation point at 25°C	4.332 Pa	Bernacchi *et al*. (2001)
ξ	Sensitivity of ratio of leaf‐internal to ambient partial pressures of CO_2_ to vapour pressure deficit	Pa^1/2^	Prentice *et al*. (2014); Wang *et al*. (2017)
φ_0_	Intrinsic quantum efficiency	mol mol^−1^	Bernacchi *et al*. (2001)
φ_0_(*T*)	Temperature dependence function of intrinsic quantum efficiency	mol mol^−1^	Bernacchi *et al*. (2001)

### Temperature response curve

We simulated the temperature response of photosynthesis using the subdaily P model by varying only the growth temperature (*T*_accl_, defined as the 15‐d mean midday temperature) while keeping all other environmental drivers constant (PAR = 1800 μmol m^−2^ s^−1^, VPD = 500 Pa, P_atm_ = 101 325 Pa, CO_2_ = 400 ppm). We adjusted growth temperature by 5°C increments from 10°C to 40°C. We generated temperature–response curves at each growth temperature by varying instantaneous leaf temperature from 0 to 60°C under the same fixed drivers.

### Leaf‐level gas exchange data

The first dataset used to compare with the simulations consisted of leaf‐level measurements from plants grown under controlled chamber conditions from the ‘ACi‐TGlob_V1.0’ dataset (Kumarathunge *et al*., 2018). The dataset includes 17 C_3_ species, representing tropical trees, temperate trees, grasses and crops (Tables 2, S1). To facilitate comparison with estimates from the subdaily P model, which separates acclimated from instantaneous responses, we only retained records that had information on both prior growth and current measurement conditions and that were grown under ambient CO_2_ with no soil water stress. Data processing followed the procedures described in Kumarathunge *et al*. (2019) with supporting data and scripts available from the ‘photom’ Bitbucket repository (https://bitbucket.org/Kumarathunge/photom). Any missing information was obtained from the original publications.

**Table 2:** Growth treatment and measurement conditions by dataset and species.

Dataset	Species	Measurement PPFD (μmol m^−2^ s^−1^)	Measurement temperature range (°C)	Growth temperature (°C)	Growth light (μmol m^−2^ s^−1^)	Growth moisture	References
Eucalyptus tereticornis provs, AU‐NSW	*Eucalyptus tereticornis* prov. temperate prov. tropical	1500	18–42	18, 28.5 and 35.5	1120	18°C – 0.7 kPa 28.5°C – 1.46 kPa 35.5°C – 2.2 kPa	Crous *et al*. (2018)
Ghannoum Eucalypt spp., AU‐NSW	*Eucalyptus saligna*, *Eucalyptus sideroxylon*	1500	15–43	26 and 30	1250	Relative humidity averaged 70% over the growing season	Ghannoum *et al*. (2010)
Smith $C_{3}$ spp., IN, USA	*Acer rubrum* *Betula alleghaniensis* *Cedrela odorata* *Elymus Canadensis* *Glycine max* *Pinus nigra* *Pinus pinaster* *Pinus pinea* *Pinus sylvestris* *Poa pratensis* *Quercus virginiana* *Tamarindus indica* *Triticum aestivum* *Ulmus americana*	1200	14–50	15, 20, 25, 30 and 35	*c*. 1470	In each case, relative humidity was set to 50%	Smith & Dukes (2017)

The *Eucalyptus tereticornis* measurements were made on trees from different provenances (temperate and tropical). The light level is measured by photosynthetic photon flux density and the growth moisture levels are measured either as vapour pressure deficit or as relative humidity. Growth light and moisture were either directly obtained from the original paper or extracted from figures using Webplotdigitizer (v.5, https://automeris.io/WebPlotDigitizer).

The studied plants were grown in a wide range of growth environments designed to allow for thermal acclimation. Growth temperatures ranged from 15°C to 35.5°C. Depending on the experimental set‐up, either VPD varied between 0.7 and 2.2 kPa, or relative humidity varied between 50% and 70%. Growth light levels were consistently high, ranging from 1120 to 1470 μmol m^−2^ s^−1^. Measurements were made under standardised high light (PPFD 1200–1500 μmol m^−2^ s^−1^) across a wide leaf temperature range of 14–50°C to capture full temperature–response curves.

For each leaf temperature, ambient *A*_net_ was taken at *c*. 400 ppm CO_2_. If the first point of the *A*–*C*_i_ curve was not measured at ambient CO_2_, we used the *A*_net_ value corresponding to measured CO_2_ concentration between 390 and 410 ppm. We only retained records that had information on both prior growth and current measurement conditions and that were grown under defined ambient CO_2_ with no soil water stress. This resulted in a dataset with 1120 observations for woody plants but only 426 observations of nonwoody species. *T*_opt_ and *A*_opt_ from data were estimated using a commonly used quadratic temperature–response model (Battaglia *et al*., 1996):

(Eqn 19) $$A_{\mathrm{net}}=A_{\mathrm{opt}}-b{\left(T_{\text{leaf}}-T_{\mathrm{opt}}\right)}^{2}$$

where *T* is in °C, *A*_opt_ is the rate at *T*_opt_, and *b* is a parameter describing the curvature (units: μmol m^−2^ s^−1^°C^−2^). These derived values of $A_{\mathrm{opt}}$ and $T_{\mathrm{opt}}$ were subsequently used to compare with the P model predictions.

Fits were obtained using nonlinear mixed‐effects models. Species were included as a random effect or, when only one species was present, the measurement replicate was used instead. To ensure quality, we retained only curves that met the following criteria: at least five temperature points, successful model convergence, SE for *A*_opt_ < 5 μmol m^−2^ s^−1^ and *T*_opt_ < 5°C, and a positive curvature coefficient (*b* > 0).

We derived *V*_cmax_ and *J*_max_ from the gas exchange measurements by fitting *A*–*C*_i_ response curves to the FvCB model using the plantecophys::fitacis function in R (Duursma, 2015). Only fitted curves with *R* ^2^> 0.99 were retained.

For each species and growth temperature treatment, the temperature response of *V*_cmax_ and *J*_max_ was subsequently fitted using the peaked Arrhenius function (Johnson *et al*., 1942):

(Eqn 20) $$f\left(T_{k}\right)=k_{25}\cdot \exp\left[\frac{E_{a}\left(T_{k}-298.15\right)}{R\cdot \left(T_{k}\cdot 298.15\right)}\right]\cdot \frac{1+\exp\left(\frac{298.15\cdot \Delta S-\mathrm{Hd}\;}{R\cdot 298.15}\right)}{1+\exp\left(\frac{T_{k}\cdot \Delta S-\mathrm{Hd}}{R\cdot T_{k}}\right)}$$

where *f*(*T*_k_) is the process rate at a given leaf temperature representing either *V*_cmax_ or *J*_max_ (μmol m^−2^ s^−1^). The function is parameterised by the corresponding process rate at 25°C ($k_{25}$), the activation energy (*E*_a_ kJ mol^−1^), an entropy term Δ*S* (kJ mol^−1^ K^−1^) and a deactivation energy (*H*_d_, kJ mol^−1^). A fixed value of 200 000 J mol^−1^ for *H*_d_ was constrained (Dreyer *et al*., 2001; Medlyn *et al*., 2002) while *k*_25_, *E*_a_ and Δ*S* for the experiments were estimated using nonlinear mixed‐effects models. For the P model simulations, *V*_cmax25_ and *J*_max25_ were estimated from environmental drivers within the 15‐d acclimation window, including the acclimation temperature, light, VPD, atmospheric CO_2_ and air pressure.

### FLUXNET site data

The second dataset included ecosystem‐scale measurements from 11 eddy covariance flux towers from the FLUXNET2015 dataset (Pastorello *et al*., 2020). The sites are distributed across North America, Europe, Asia and Australia and represent a range of climate and vegetation types (Tables 3, S2). All sites are in humid climates, with mean annual temperature (MAT) ranging from −3.2°C (CA‐Man) to 10.7°C (AU‐Tum) and mean annual precipitation (MAP) ranging from 429 mm (CA‐Oas) to 1159 mm (AU‐Tum).

**Table 3:** Information on the eddy covariance flux tower sites.

SiteID	Latitude	Longitude	IGBP	Aridity	MAT (°C)	MAP (mm)
AU‐Tum	−35.657	148.152	EBF	Humid	10.7	1159
US‐Ha1	42.5378	−72.172	DBF	Humid	6.6	1071
US‐UMB	45.5598	−84.714	DBF	Humid	5.8	803
CH‐Cha	47.2102	8.4104	GRA	Humid	9.5	1136
BE‐Vie	50.3049	5.9981	MF	Humid	7.8	1062
DE‐Gri	50.95	13.5126	GRA	Humid	8.4	877
DE‐Hai	51.0792	10.4522	DBF	Humid	8.34	744
CA‐Oas	53.6289	−106.2	DBF	Humid	0.34	429
CA‐Man	55.8796	−98.481	ENF	Humid	−3.2	520
RU‐Fyo	56.4615	32.9221	ENF	Humid	3.9	711
FI‐Hyy	61.8474	24.2948	ENF	Humid	3.8	709

The SiteID is from the FLUXNET2015 dataset (Pastorello *et al*., 2020). Latitude and longitude are given in decimal degrees, in which positive values are north and east, respectively, and negative values are south and west, respectively. The International Geosphere and Biosphere Progamme (IGBP) vegetation codes are evergreen broadleaf forest, deciduous broadleaf forest (DBF), mixed forest (MF), evergreen needleleaf forest (ENF), and grassland (GRA). As indicated by the aridity column, all sites are considered humid. Site estimates of mean annual temperature (MAT) and mean annual precipitation (MAP) are given.

We restricted the analysis to the growing season, defined as the period when daily mean air temperature was above 5°C and the fraction of absorbed photosynthetically active radiation (fAPAR) was > 0.3. We applied a 15‐d moving window to represent the acclimation timescale and calculated the growth temperature (*T*_growth_), approximated as *T*_air_ in this comparison, as the average midday air temperature within this 15‐d window consistent with the approach used in the subdaily P model (‘The subdaily P model’ in the Materials and Methods section).

Observed GPP was taken from the daytime partitioned GPP (GPP_DT_CUT_REF) provided by FLUXNET2015 (Pastorello *et al*., 2020).

To obtain simulated GPP, we used site‐level meteorological observations to drive the subdaily P model, including incoming shortwave radiation (SW_IN_F), air temperature (TA_F), vapour pressure deficit (VPD_F) and atmospheric pressure (PA_F). We calculated fAPAR using Beer's law (Beer *et al*., 2010), fAPAR = 1 – exp (−k · LAI) with *k* = 0.5, based on Leaf Area Index (LAI) data obtained from the MODIS MCD15A3H product (Myneni *et al*., 2015). The sites are distributed across North America, Europe, Asia and Australia and represent a range of climate and vegetation types (Tables 3, S2). Nine of these sites provide information on a half‐hourly timestep, but two of the sites (US‐UMB and US‐Ha1) only provide data on an hourly timestep, which was interpolated to half‐hourly resolution for consistency. Observed and modelled *A*_opt_ and the *T*_opt_ was calculated in the same way: The maximum half‐hourly (or hourly) GPP in each 15‐d window was identified as *A*_opt_, and the corresponding temperature at that time was taken as *T*_opt_.

### Statistical analysis

For the gas exchange dataset, *T*_opt_ and the *J*_max25_/*V*_cmax25_ ratio were related to *T*_growth_ using the Standard Major Axis (SMA) regression within each Dataset × Species group, while *A*_opt_ (normalised by the group‐specific maximum) was analysed using quadratic regression. Analyses were conducted separately for woody and nonwoody species. For the FLUXNET dataset, linear regression was fitted to *T*_opt_ vs *T*_growth_ and quadratic regression to *A*_opt_norm_, where *A*_opt_ was normalised by the maximum *A*_opt_ within each calendar year at that site. SMA regressions were made using the smatr package (Warton *et al*., 2012), and quadratic and mixed‐effects regression models were fitted using the nlme package (Pinheiro *et al*., 2025). Fitting was performed separately for observed GPP and for P model GPP. The goodness of fit was summarised by *R* ^2^ and Root Mean Square Error (RMSE). The agreement between the modelled and observational trend was further evaluated using RMSE_imp. RMSE_imp is calculated by applying the modelled regression line to the observational data and therefore provides a direct measure of how well the modelled trend matches the observed data.

## Results

**Fig. 2:**
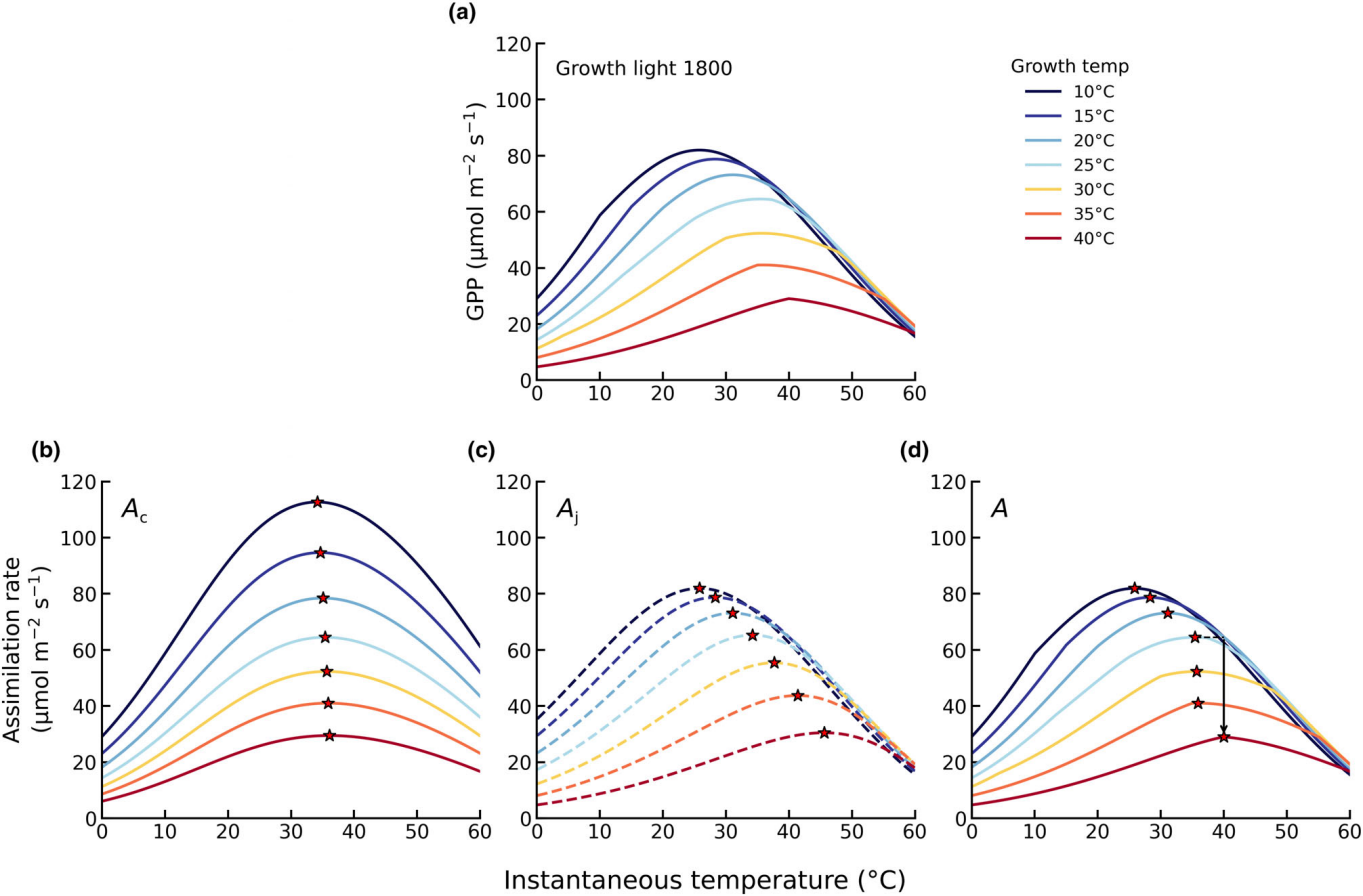
Simulated temperature responses of (a) gross primary production, (b) $A_{c}$, (c) $A_{j}$ and (d) *A*. Lines are coloured by growth temperature. Red stars indicate the temperature at which assimilation reaches its maximum. Open circles mark the transition points where limitation shifts between $A_{c}$ and $A_{j}$ and the dashed line with a downward arrow illustrates the shift in the thermal optimum as growth temperature increases from 25°C to 40°C.

The simulated response of *T*_opt_ under high‐light conditions (1800 μmol photons m^−2^ s^−1^) increased with growth temperature shifting from *c*. 25°C at a growth temperature of 10°C to *c*. 38°C at a growth temperature of 40°C (Fig. 2a). *T*_opt_ was considerably higher than *T*_growth_ at lower growth temperatures, but the difference became smaller as growth temperature increased. This indicates that photosynthesis operates further from the thermal optimum under cooler conditions and closer to the thermal optimum under warmer growth conditions. *A*_opt_ decreased with increasing growth temperature, with a steeper decline observed at higher growth temperatures. Across growth temperature of 25–40°C, *A*_opt_ decreased by more than 50% between the lowest and highest growth temperatures. At instantaneous temperatures above 50°C, GPP declined strongly across all growth temperatures. Additional experiments in which vapour pressure (VP) was held constant but both growth and instantaneous VPD were allowed to vary through the temperature dependence of saturation VP showed the same overall pattern, indicating that VPD changes do not alter the main trends in the temperature response of GPP (Fig. S1). Modest increases in c_i_ occur across the temperature range considered (Fig. S2a), particularly under lower growth temperature conditions. Larger changes in c_i_ (declining, then increasing) are shown in the experiment in which VPD was allowed to vary with temperature.

The temperature responses of *A*_c_ (Fig. 2b) and *A*_j_ (Fig. 2c) change with growth temperature. The peak assimilation rate of *A*_c_ decreased with increasing growth temperature, and the temperature at which the peak occurred ranged between 34°C and 36°C. The peak rate of *A*_j_ also declined, but its temperature optimum shifted substantially, increasing from *c*. 25°C at low growth temperatures to *c*. 45°C at high growth temperatures. Depending on growth temperature, the *T*_opt_ of *A* occurred either in the *A*_c_‐limited region or in the *A*_j_‐limited region (Fig. 2d). For example, at a growth temperature of 15°C, the *T*_opt_ was in the *A*_j_‐limited region, whereas at 35°C, it was located in the *A*_c_‐limited region. These results highlight that the *T*_opt_ of GPP is not determined by a single process but emerges from the shifting balance between carboxylation and electron transport limitations, as well as changes in the shape of these temperature–response curves under different growth conditions.

The shift in the optimum temperature of *A*_j_ closely matched that of *J* (*T*_opt,J_), reflecting the direct dependence of *A*_j_ on the electron transport rate (Fig. S3). We examined the factors controlling the acclimated temperature response of *J*(T) primarily driven by changes in *J*_max25_. As growth temperature increases, *J*_max25_ declines rapidly (Fig. S4). A lower *J*_max25_ reduces the capacity for electron transport at a given instantaneous temperature (Fig. S5), which in turn affects the balance between light limitation and capacity limitation in the calculation of *J*(T).

The position of *T*_opt,J_ is therefore not determined directly by the absolute value of *J*_max25_, but rather by the balance point between light‐driven and capacity‐driven processes. A lower *J*_max25_ extends the dominance of A_j_, and the eventual switch to A_c_ only occurs at higher instantaneous temperatures, resulting in a higher *T*_opt,J_.

On the other hand, the small shift in optimum temperature of *A*_c_ (*T*_opt,Ac_) is primarily driven by changes in $\frac{C_{i}-\Gamma^{*}}{C_{i}+K}$. The shape of the *V*_cmax_ temperature response is unchanged and mainly reflects acclimation of ξ (Fig. S6). When *c*_i_ acclimates through changes in ξ, the decline of $\frac{C_{i}-\Gamma^{*}}{C_{i}+K}$ with increasing temperature becomes slightly less steep. As a result, higher *c*_i_ at higher growth temperature (Fig. S6) leads to a weaker temperature‐driven decrease in $\frac{C_{i}-\Gamma^{*}}{C_{i}+K}$, which in turn causes a slight rightward shift in *T*_opt,Ac_ (Fig. 2b).

**Fig. 3:**
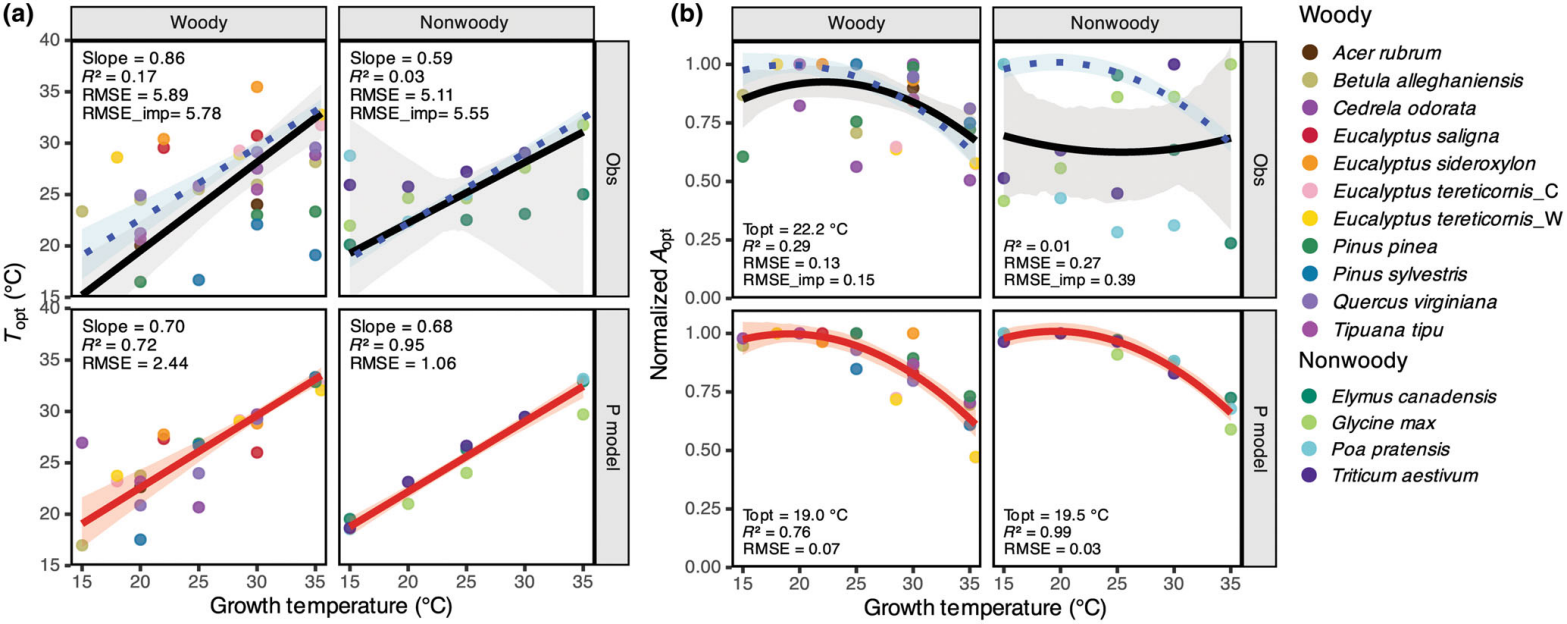
Observed vs modelled relationships of (a) *T*_opt_ and growth temperature and (b) Normalised *A*_opt_ and growth temperature for woody and nonwoody species. In the upper panels, points represent values estimated from observations through curve fitting. Black solid lines represent regression fits to observed data and the grey‐shaded area corresponds to the 95% confidence intervals. Blue dotted lines show the P model imposed fits and are the same as those shown in the bottom panels, and the blue‐shaded area corresponds to the 95% confidence intervals. In the lower panels, points represent values derived from P model simulations driven by the observed environmental conditions. Red solid lines represent P model fits, and the red shaded areas indicate the 95% confidence intervals. *Eucalyptus tereticornis*_C and *Eucalyptus tereticornis*_W correspond to trees from temperate and tropical provenances, respectively.

The relationship between growth temperature and photosynthetic temperature–response traits was examined for woody and non‐woody species using both experimental data and modelled outputs (Fig. 3). The observations show a strong increase in *T*_opt_ with *T*_growth_ in woody species (slope = 0.86, RMSE = 5.89°C; Fig. 3a). However, the relationship for non‐woody species was weak and nonsignificant (slope = 0.59, RMSE = 5.11°C; Fig. 3a). The subdaily P model produced a consistent positive relationship for both plant types (Fig. 3a). The modelled slope was 0.70 for woody species, with a high *R* ^2^ of 0.72 and a low RMSE of 2.44°C. For non‐woody species, the model predicted a similar slope (0.68) with an *R* ^2^ of 0.95 and a low RMSE (1.06°C), indicating a strong and consistent acclimation response in the model. The contrast between the clear modelled response and the absence of a significant observational trend for non‐woody species is not a reflection of the tendency for the P model to overestimate the response but likely reflects the limited sample size (426 observations).

The observed relationship between normalised *A*_opt_ and *T*_growth_ (Fig. 3b) showed a peaked response for woody species, with an optimum at 22.2°C (*R* ^2^ = 0.29) beyond which photosynthetic capacity declined. This shows that there is a threshold beyond which growth temperature begins to limit photosynthetic capacity. There was no significant peaked relationship for nonwoody species. The model reproduced this peaked relationship for both plant types, with a similar optimum of 19°C for woody and an optimum of 19.5°C for nonwoody species. The model fit was particularly strong for both woody species (*R* ^2^ = 0.76) and nonwoody species (*R* ^2^ = 0.99). For woody species, RMSE_imp was similar to the observational RMSE for both *T*_opt_ (RMSE = 5.89°C and RMSE_imp = 5.78°C) and normalised *A*_opt_ (RMSE = 0.13 and RMSE_imp = 0.15), indicating that the modelled trend closely matched the observed pattern.

**Fig. 4:**
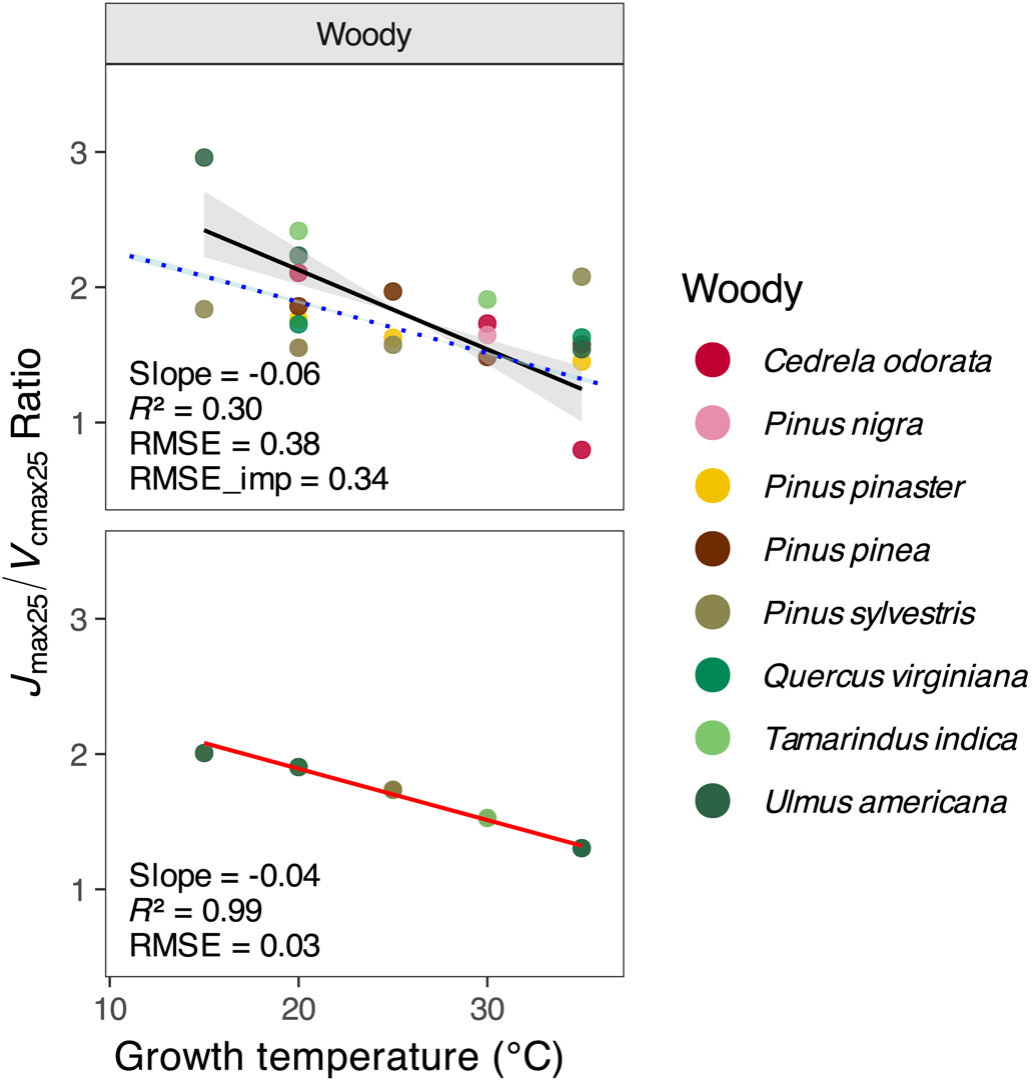
Observed and modelled relationships between *J*_max25_/*V*_cmax25_ ratio and growth temperature for woody species. Points represent values derived from observations (upper panel) and P model simulations driven by observed environmental conditions (lower panel). Black solid lines represent regression fits to observed data, and blue dashed lines show P model imposed fits. Red solid lines represent P model fits. Grey‐, blue‐ and red‐shaded areas indicate the 95% confidence intervals for observed, imposed and P model fits, respectively.

The observed and modelled ratio of *J*_max25_ to *V*_cmax25_ in woody species declined with increasing *T*_growth_ (Fig. 4). Both the observed and modelled *J*_max25_/*V*_cmax25_ ratios showed a negative relationship with growth temperature. The observed trend was weak (slope = −0.06, *R* ^2^ = 0.30) and variable across species, whereas the P model produced a much stronger relationship (slope = −0.04, *R* ^2^ = 0.99) with low scatter (RMSE = 0.03). The RMSE of the observational fit (0.38) was close to that of the imposed model regression (0.34). These results suggest that both the model and observations are consistent in showing a decline in *J*_max25_/*V*_cmax25_ with warming, although the observations show greater variability across species.

Both the P model and observations showed a positive relationship between *T*_opt_ of GPP and *T*_growth_ across all flux tower sites (Table 4). The model predicted that T_opt_ increased by 0.72–1.13°C for each 1°C increase in *T*_growth_ across the sites. The observed responses were similar, with *T*_opt_ increasing by 0.75–1.07°C for every 1°C rise in growth temperature. While the direction of change was consistent, the magnitude of the response differed between sites. At most sites, the P model produced a slightly higher rate of increase in *T*_opt_ than that of the observations, for example at FI‐Hyy (model 1.10 vs obs 0.86), CA‐Man (1.08 vs 0.96), CA‐Oas (0.99 vs 0.75), DE‐Hai (0.98 vs 0.78), BE‐Vie (1.13 vs 0.86) and CH‐Cha (1.02 vs 0.88).

**Table 4:** Summary of site‐level relationships between *T*_opt_, normalised *A*_opt_ and *T*_growth_ for model and observations (*T*_opt_: Slope, *R* ^2^ and RMSE; *A*_opt_: *R* ^2^ and RMSE).

Site	Var	Model_slope	Obs_slope	Model_*R* ^2^	Model_SD	Obs_*R* ^2^	Obs_SD	Imposed_SD
AU‐Tum	*T*_opt_	1.06	1.07	0.88	1.88	0.77	2.79	2.79
BE‐Vie	*T*_opt_	1.13	0.86	0.84	2.45	0.71	2.75	3.08
CA‐Man	*T*_opt_	1.08	0.96	0.74	3.43	0.55	4.67	4.71
CA‐Oas	*T*_opt_	0.99	0.75	0.81	3.02	0.77	2.60	3.02
CH‐Cha	*T*_opt_	1.02	0.88	0.88	2.47	0.73	3.47	3.59
DE‐Gri	*T*_opt_	1.04	1.03	0.85	2.50	0.77	3.20	3.20
DE‐Hai	*T*_opt_	0.98	0.78	0.77	2.52	0.66	2.61	2.77
FI‐Hyy	*T*_opt_	1.10	0.86	0.85	2.46	0.80	2.26	2.58
RU‐Fyo	*T*_opt_	0.86	0.79	0.75	2.61	0.70	2.65	2.67
US‐Ha1	*T*_opt_	0.95	0.99	0.81	2.69	0.77	3.21	3.23
US‐UMB	*T*_opt_	0.72	0.84	0.63	2.59	0.57	3.43	3.47
AU‐Tum	*A*_opt_			0.85	0.07	0.41	0.11	0.13
BE‐Vie	*A*_opt_			0.84	0.11	0.77	0.12	0.13
CA‐Man	*A*_opt_			0.35	0.17	0.21	0.28	0.31
CA‐Oas	*A*_opt_			0.65	0.15	0.80	0.17	0.22
CH‐Cha	*A*_opt_			0.78	0.11	0.72	0.11	0.12
DE‐Gri	*A*_opt_			0.81	0.11	0.72	0.14	0.14
DE‐Hai	*A*_opt_			0.80	0.11	0.76	0.14	0.15
FI‐Hyy	*A*_opt_			0.82	0.11	0.84	0.10	0.10
RU‐Fyo	*A*_opt_			0.81	0.10	0.70	0.12	0.13
US‐Ha1	*A*_opt_			0.80	0.11	0.78	0.12	0.14
US‐UMB	*A*_opt_			0.66	0.10	0.71	0.13	0.15

The specific years used for each site and variable are listed in Supporting Information Table S2.

**Fig. 5:**
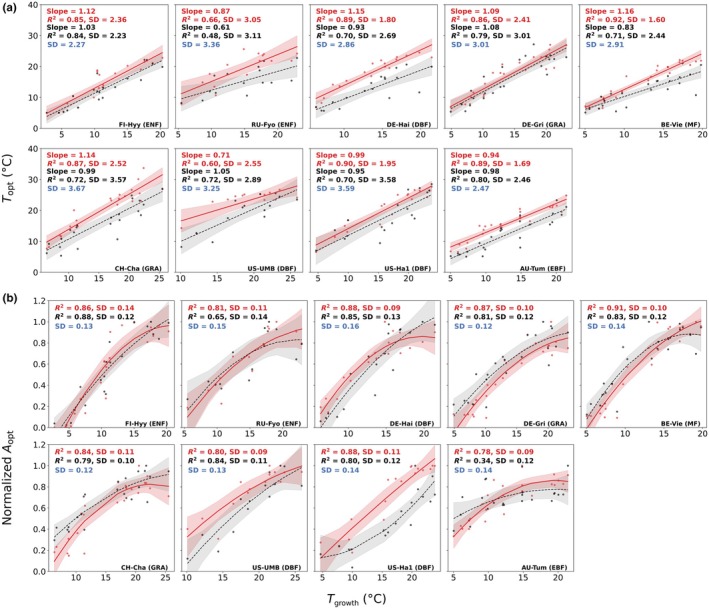
Relationships between (a) *T*_opt_ and (b) normalised *A*_opt_ with growth temperature across sites in 2007. Black points, black labels, and black‐dashed lines represent observations. Red points, red labels, and red solid lines represent subdaily P model predictions, while blue labels indicate results from the imposed simulation. Shaded areas represent the 95% confidence intervals for the fitted regressions.

The P model tends to overestimate the *T*_opt_–*T*_growth_ relationship, with predicted *T*_opt_ values generally higher than observed across growth temperatures at most sites (Fig. 5a). This pattern remained consistent throughout all simulation years in the analysis (Fig. S7). However, the imposed SD was very close to the observed SD (e.g. DE‐Gri: imposed 3.01 vs observed 3.01; AU‐Tum: imposed 2.47 vs observed 2.46). This close match indicates that the scatter around the relationship was similar, regardless of whether it was based on the model or observational fit. The similarity in SD suggests that the observed relationship between *T*_opt_ and *T*_growth_ is captured by the P model.

Both the P model and observations showed a peaked relationship between normalised *A*_opt_ and growth temperature across all sites (Table 4; Fig. 5b). In most sites, normalised *A*_opt_ increased with growth temperature up to an optimum and then declined at higher temperatures, consistent with the trend shown in the gas exchange experiments.

We tested the impact of using *T*_air_ rather than *T*_leaf_ at the FLUXNET sites by running sensitivity analyses in which we imposed a difference between *T*_leaf_ and *T*_air_ (Δ*T*) ranging from −2 to +8°C, a range consistent with leaf‐level and canopy‐level observations (Linacre, 1964; Michaletz *et al*., 2016; Dong *et al*., 2017; Still *et al*., 2022; Manzi *et al*., 2024) with VP fixed but allowing VPD to increase naturally with temperature over a range of baseline *T*_air_ temperatures from 10 to 40°C. The results show that substituting *T*_leaf_ for *T*_air_ would have negligible impact on the results (Fig. S8; Table S3) at the FLUXNET sites, which generally have growing season temperatures below 25°C (Fig. 5) and are located in relatively well‐watered regions.

## Discussion

The generally good agreement between simulated and observed A–T relationships shows that the subdaily P model captures the emergent temperature dependence of photosynthetic parameters. The model successfully reproduces the observed increase in *T*_opt_ with growth temperature, along with the coordinated changes in *A*_opt_ and the decline in *J*_max25_/*V*_cmax25_. This agreement shows that the processes embedded in the model, specifically dynamic adjustment of the *J*_max25_ */V*_cmax25_ ratio and stomatal behaviour, are sufficient to explain observed acclimation to temperature. Thus, the model provides an explanation of how plants balance carboxylation and electron transport limitations under varying thermal growing conditions.

The analyses at flux tower sites of necessity used *T*_air_ as a surrogate for *T*_leaf_. Observations show that leaf temperatures can be cooler than the air under hot but well‐watered conditions and warmer than the air in cool environments and in warm environments where high radiation and high VPD limits transpirational cooling (Linacre, 1964; Michaletz *et al*., 2016; Dong *et al*., 2017; Still *et al*., 2022; Manzi *et al*., 2024). Sensitivity analyses show that increasing or decreasing the *T*_air_ by a range consistent with plausible differences between *T*_air_ and *T*_leaf_ (Δ*T*) show that this substitution would have little impact on the estimate of *T*_opt_ except when *T*_growth_ was above 30°C or Δ*T* was very large (8°C). Most of the FLUXNET sites have growing season temperatures below 25°C and are located in relatively well‐watered regions and thus it is unlikely that using *T*_air_ could significantly biases the estimation of *T*_opt_.

Ecosystem‐scale estimates of *T*_opt_ and *A*_opt_ from flux towers are not directly comparable with leaf‐level measurements. Flux data integrate responses across whole canopies and varying environmental conditions, while the experimental data reflect individual leaf responses under controlled settings. However, the observations were broadly consistent at both scales, indicating that acclimation can be observed at leaf level and at ecosystem level. Indeed, other studies have reported ecosystem‐level acclimation (Liu *et al*., 2024; Schneider *et al*., 2025). Liu *et al*. (2024) examined canopy‐scale acclimation of photosynthesis using light–response curves across the FLUXNET2015 dataset to derive maximum photosynthetic rate across a range of temperatures (*T*_air_) and vegetation types. Using different configurations of the Breathing Earth System Simulator model (BESS), they showed that acclimation was necessary in order to obtain a realistic range of sensitivities to *T*_air_. The average timescale of acclimation was ca 14 d, consistent with the assumption used in the subdaily P model scheme. The average rate of thermal acclimation varied depending on vegetation type, but the range is consistent with our findings. They did not consider how *A*_opt_ varied with *T*_growth_, nor explain the negative sensitivity to *T*_air_ that was shown at high temperatures. Schneider *et al*. (2025) analysed the contributions of different modelled acclimatory processes and compared them to the same experimental dataset. They highlighted the potential of optimality principles in capturing acclimation trends across environmental gradients, and concluded that responses of stomatal sensitivity, photosynthetic capacities and enzyme kinetic parameters are all needed in order to explain the observed increases of *T*_opt_ with growth temperature. However, our P model implementation (identical with Mengoli *et al*., 2022), which includes the stomatal sensitivity and photosynthetic capacity responses but no adjustment of enzyme kinetics, is shown here to reproduce both the increase of *T*_opt_ and the nonlinear decline of normalised *A*_opt_ with growth temperature (which Schneider *et al*. (2025) did not discuss). More detailed comparison is impossible because their implementation depends on formulations of *A*_J_ and *V*_cmax_ that deviate in several respects from the standard FvCB model as applied here.

Both the experimental and flux tower observations could be affected by the covariation of VPD and temperature. However, in our analysis, we kept VPD constant when fitting the modelled temperature response curves (Fig. 2). The overall similarity between the model and observations suggests that temperature is the main factor driving the observed patterns. In the VPD sensitivity analysis (Fig. S1), while air temperature varied, the ambient VP was fixed at 500 Pa, meaning that VPD(*T*) = *e*_s_(*T*) – 500. Here, *e*_s_(*T*) represents the saturation VP at temperature *T*. This configuration keeps absolute humidity constant and allows VPD to increase with *T*, which is similar to the ‘constant dewpoint’ scenario used by For the gas exchange dataset, *T*_opt_ and the *J*_max25_/*V*_cmax25_ ratio were related to *T*_growth_ using SMA regression within each Dataset × Species group, while *A*_opt_ (normalised by the group‐specific maximum) was analysed using quadratic regression. Analyses were conducted separately for woody and non‐woody species. Additional tests in which VPD was allowed to vary with temperature produced broadly similar GPP responses to those obtained when VPD was held constant (Fig. S1). This similarity in GPP responses suggests that the response is primarily temperature‐driven and is not substantially modified by the covariation of temperature and VPD.

Empirical schemes (e.g. Kattge & Knorr, 2007; Kumarathunge *et al*., 2019) allow the activation energy (*E*_a_), deactivation energy (*H*_d_), and entropy term (Δ*S*) in the peaked Arrhenius function to vary as a function of growth temperature in order to adjust the temperature–response curve parameters of *V*_cmax_ and *J*_max_; capture how plants grown at higher temperatures exhibit a less negative Δ*S*, which delays deactivation at high temperatures and increases *T*_opt_. Nevertheless, while these empirical approaches improve the description of temperature effects, they rely on empirical parameter fitting rather than mechanistic representation of physiological processes (Mercado *et al*., 2018; Smith & Keenan, 2020). In the subdaily P model framework, changes in *T*_opt_ arise from the environmental control of *J*_max25_/*V*_cmax25_, specifically growth temperature, light, VPD and CO_2_, and thus do not require calibration of extra parameters.

We have focussed on temperature as the primary driver of acclimation. However, plants in natural environments are simultaneously exposed to other changing factors. Understanding these additional influences is important for placing temperature‐driven acclimation in a broader ecological context. Observations from experiments and field studies indicate that plants adjust their photosynthetic traits not only to temperature (Campbell *et al*., 2007; Drake *et al*., 2015; Crous *et al*., 2022, 2025; Dusenge *et al*., 2024) but also to light, atmospheric CO_2_ concentration and VPD. Acclimation to light availability has been reported in diverse ecosystems (Bachofen *et al*., 2020; Luo & Keenan, 2020). Long‐term CO_2_ enrichment experiments show reductions in *V*_cmax_ and *J*_max_ under elevated CO_2_ (Yang *et al*., 2020; Smith *et al*., 2024). VPD has also been identified as a factor influencing photosynthetic capacity and stomatal conductance (Marchin *et al*., 2016; López *et al*., 2021; Middleby *et al*., 2024). These findings indicate that acclimation reflects the combined influence of growth temperature, light, VPD and CO_2_.

The standard version of the P model (Prentice *et al*., 2014; Wang *et al*., 2017; Stocker *et al*., 2020) captures both the broad‐scale spatial and seasonal patterns in photosynthetic traits as well as GPP (Smith *et al*., 2019; Dong *et al*., 2023; Cai *et al*., 2025). In comparison with the 15 dynamic global vegetation models used in the ‘Trends and drivers of the regional scale terrestrial sources and sinks of carbon dioxide’ (TRENDY) project (Sitch *et al*., 2015, 2024), the model has been shown to simulate both the spatial patterns and the recent trends in the seasonal maximum fAPAR as well as the four best models, and achieves the highest overall R^2^ of all the models (Cai *et al*., 2025). The offline version of the subdaily P model has been shown to be better at capturing diurnal and seasonal variability in GPP than the European Centre for Medium‐Range Weather Forecasts LSM (Mengoli *et al*., 2022), and a fully coupled version of this model has been shown to substantially improve GPP predictions in Noah‐MP (Ren *et al*., 2025). Nevertheless, both the original form of the model and the subdaily version overestimate GPP under drought conditions. A number of empirical corrections have been developed to account for drought stress by applying a soil moisture stress factor directly to GPP. Drought effects are represented by multiplying well‐watered GPP by a soil moisture stress factor β(θ), defined as a function of plant‐available soil moisture that increases with θ, reaches unity at a fixed critical threshold θ*, and remains constant for soil moisture values above this threshold (Stocker *et al*., 2020). Mengoli *et al*. (2025) extended this framework using breakpoint regression to show that the critical threshold θ* and the sensitivity of β(θ) vary systematically with climatic aridity, allowing the soil moisture response to adjust across ecosystems. Although these corrections affect GPP rather than modulating the stomatal response explicitly, they improved P model predictions of GPP in dry regions. Incorporating drought responses within the P model framework would require coupling stomatal conductance more directly to the soil water balance and relaxing the assumption of a constant unit cost of transpiration, allowing this cost to vary dynamically with rooting‐zone soil moisture availability. In the current version of the model, we assume that the quantum efficiency of photosynthesis at given temperature (φ_0_) is constant. This is known to be an oversimplification (Sandoval *et al*., 2023) and incorporating a more realistic treatment of the variation of φ0 with environmental conditions could further improve the realism of the P model simulations.

Our model predicts an upward shift in *T*_opt_ with warming but does not impose an upper limit on this shift because it currently does not consider the biochemical and biophysical constraints on photosynthetic metabolism. Increases in Rubisco activity and electron transport can sustain higher photosynthetic rates only up to the point where thermal deactivation of key enzymes and imbalances in energy supply begin to occur (Scafaro *et al*., 2023). The subdaily P model does not include the effect of high‐temperature inhibition of enzyme activities. This is likely the explanation for why the model predicts a larger increase in *T*_opt_ than observed at flux tower sites. There are models that account for declining enzyme activity at higher temperatures, including the peaked Arrhenius equation (Medlyn *et al*., 2002), and this could provide a useful basis for including high‐temperature inhabitation in an EEO modelling framework. The upper thermal limit of photosynthetic function is also closely linked to the stability of photosystem II (PSII). Global analyses have shown that PSII heat tolerance averages *c*. 50°C, with an upper bound between 60°C and 66°C (O'Sullivan *et al*., 2017; Lancaster & Humphreys, 2020; Posch *et al*., 2025). The irreversible thermal damage observed in PSII under extreme heat is not considered in the current version of the subdaily P model, which therefore is likely to overestimate photosynthetic performance near the thermal limit in which PSII begins to lose functional stability. Incorporating these physiological limits into the model framework would provide a more realistic representation of the temperature ceiling for photosynthetic acclimation.

### Conclusion

The acclimation of photosynthesis to growth temperature, shown in both observations at leaf and ecosystem level and in subdaily P model simulations is a result of the dynamic adjustment of *V*_cmax25_ and *J*_max25_, and stomatal behaviour. Since the subdaily P model captures the emergent behaviour of plants in response to changes in environmental conditions, it provides a way of incorporating acclimation in land surface models without the need for empirical functions for individual plant functional types. This should, in turn, improve the reliability of predictions of changes in GPP and consequent carbo cycle feedback under future climate change.

## Competing interests

None declared.

## Author contributions

WG, ICP and SPH developed the analytical framework. MB suggested the investigation of the decline of *A*_opt_ with *T*_growth_. NA made the first analyses using flux tower data. WG conducted the analyses presented in this paper. WG, ICP, SPH, MB and PLG all contributed to the interpretation of the analyses. WG and SPH wrote the first draft of the paper, and all authors contributed to the final draft.

## Disclaimer

The New Phytologist Foundation remains neutral with regard to jurisdictional claims in maps and in any institutional affiliations.

## Supporting information


**Fig. S1** Temperature response curve of GPP under different growth temperatures, with VPD varying with temperature.
**Fig. S2** Temperature response of *c*_i_ acclimation under different growth temperatures.
**Fig. S3** Temperature response curves of *J* and *m*_j_ under different growth temperatures.
**Fig. S4** Temperature responses of *V*_cmax25_, *J*_max25_and *J*_max25_ */V*_cmax25_.
**Fig. S5** Temperature response of *J*_max_ under different growth temperatures.
**Fig. S6** Temperature responses of *V*_cmax_ and *m*_c_.
**Fig. S7** *T*_opt_ and normalised *A*_opt_ vs growth temperature across all sites and simulation years.
**Fig. S8** Effects of leaf–air temperature differences (Δ*T*) on the optimum temperature and GPP–temperature responses.
**Notes S1** Interpretation of the coordination hypothesis as an optimality criterion.
**Notes S2** Derivation of the optimal stomatal ratio χ and the sensitivity parameter ξ.
**Notes S3** Derivation of *V*_cmax_ and *J*_max_.
**Table S1** Description of the gas exchange measurement dataset.
**Table S2** Description of the FLUXNET dataset.
**Table S3** Summary of Δ*T* scenarios and corresponding changes in modelled photosynthetic optimum temperature (*T*_opt_leaf_) across growth air temperatures (*T*_growth_air_).
Please note: Wiley is not responsible for the content or functionality of any Supporting Information supplied by the authors. Any queries (other than missing material) should be directed to the *New Phytologist* Central Office.

## Data Availability

The data and code that support the findings of this study are openly available from https://zenodo.org/records/18817645. P model code is available at https://zenodo.org/records/10927149. The experimental data are available from https://figshare.com/articles/dataset/ACi-TGlob_V1_0_A_Global_dataset_of_photosynthetic_CO2_response_curves_of_terrestrial_plants_/7283567. The FLUXNET2015 data are available from https://fluxnet.org/data/fluxnet2015-dataset/.
