# Supplementary material for: An eco‐evolutionary optimality model explains the acclimated temperature response of photosynthesis
Source: New Phytol. 2026 Apr 2;250(5):2884–99. doi: 10.1111/nph.71062 (PMC13150314; doi:10.1111/nph.71062)
Supplement: Supplementary file 1 — Fig. S1 Temperature response curve of GPP under different growth temperatures, with VPD varying with temperature. Fig. S2 Temperature response of c i acclimation under different growth temperatures. Fig. S3 Temperature response curves of J and m j under different growth temperatures. Fig. S4 Temperature responses of V cmax25, J max25and J max25 /V cmax25. Fig. S5 Temperature response of J max under different growth temperatures. Fig. S6 Temperature responses of V cmax and m c. Fig. S7 T opt and normalised A opt vs growth temperature across all sites and simulation years. Fig. S8 Effects of leaf–air temperature differences (ΔT) on the optimum temperature and GPP–temperature responses. Notes S1 Interpretation of the coordination hypothesis as an optimality criterion. Notes S2 Derivation of the optimal stomatal ratio χ and the sensitivity parameter ξ. Notes S3 Derivation of V cmax and J max. Table S1 Description of the gas exchange measurement dataset. Table S2 Description of the FLUXNET dataset. Table S3 Summary of ΔT scenarios and corresponding changes in modelled photosynthetic optimum temperature (T opt_leaf) across growth air temperatures (T growth_air). Please note: Wiley is not responsible for the content or functionality of any Supporting Information supplied by the authors. Any queries (other than missing material) should be directed to the New Phytologist Central Office. [file NPH-250-2884-s001.pdf]

New Phytologist Supporting Information

Article title: An eco-evolutionary optimality model explains the acclimated temperature response of photosynthesis

Authors: Wenyao Gan[1], Nabil Alizadeh[2], Martin Best[3], Pier Luigi Vidale[4], I. Colin Prentice[2], Sandy P. Harrison[1]

1: Department of Geography and Environmental Science, University of Reading, Reading, RG6 6AB, UK

2: Department of Life Sciences, Georgina Mace Centre for the Living Planet, Imperial College London, Silwood Park Campus, Buckhurst Road, Ascot, SL5 7PY, UK

3. Meteorological Office, Fitzroy Road, Exeter, EX1 3PB, UK

4: Department of Meteorology, University of Reading, Reading, RG6 6AB, UK

Article acceptance date: 13 February 2026

The following Supporting Information is available for this article:

Fig. S1 Vapour pressure (VP) was held constant at 500 Pa for both the acclimation period and the instantaneous simulations. Thus, both growth VPD and instantaneous VPD were allowed to vary only through the temperature dependence of saturation vapour pressure.

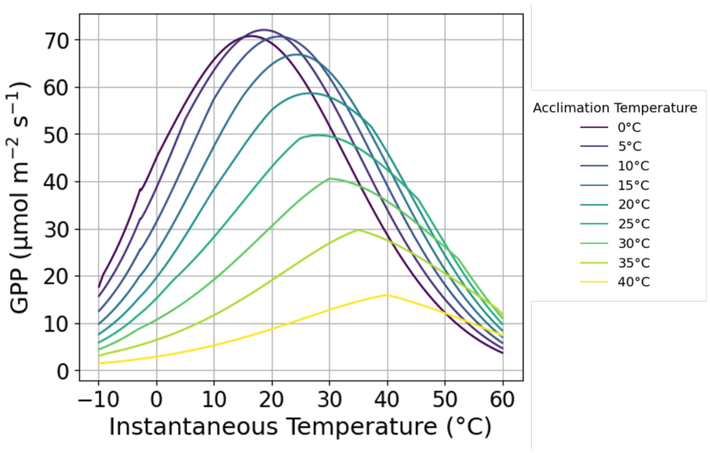

Fig. S2 Temperature response of c_i acclimation under different growth temperatures. (a) vapour pressure deficit (VPD) fixed to 500 Pa (b) VPD varies with temperature with VP fixed to 500 Pa.

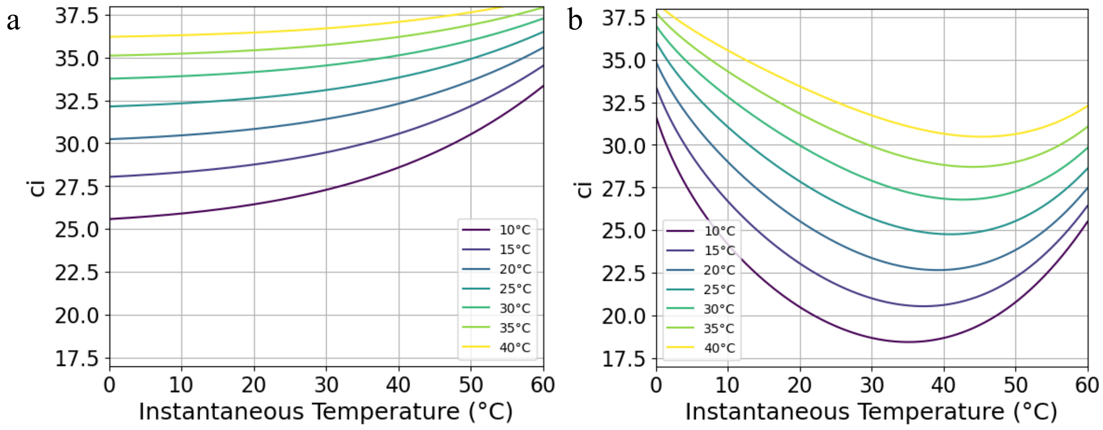

Fig. S3 Temperature response curves of J and m_j under different growth temperatures. ($m_j = \frac{(C_i - \Gamma^*)}{(C_i + 2\Gamma^*)}$

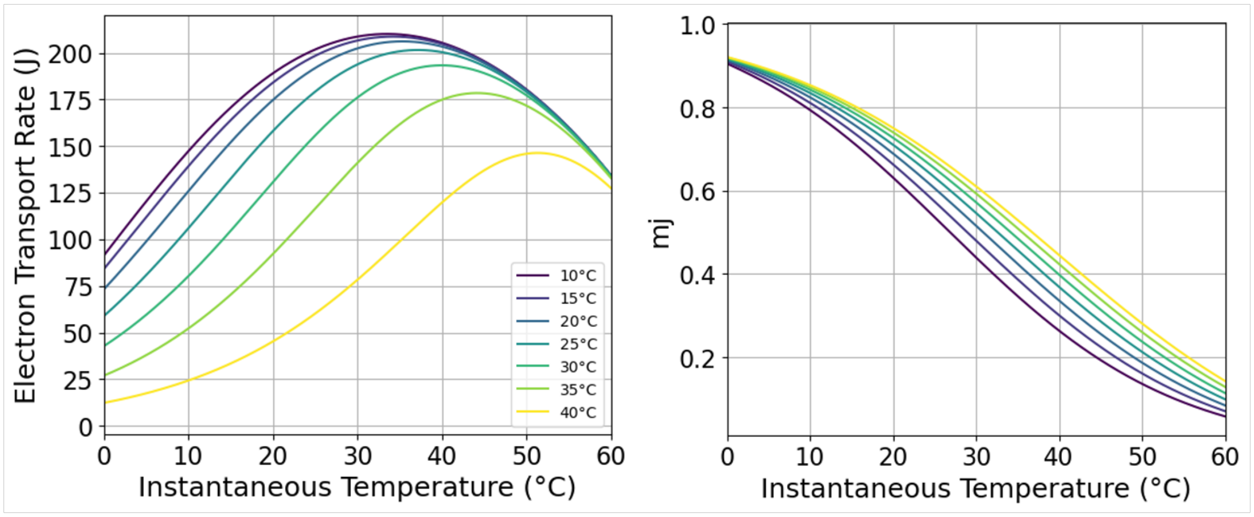

Fig. S4 Temperature responses of V_{cmax25}, J_{max25} and J_{max25}/V_{cmax25}.

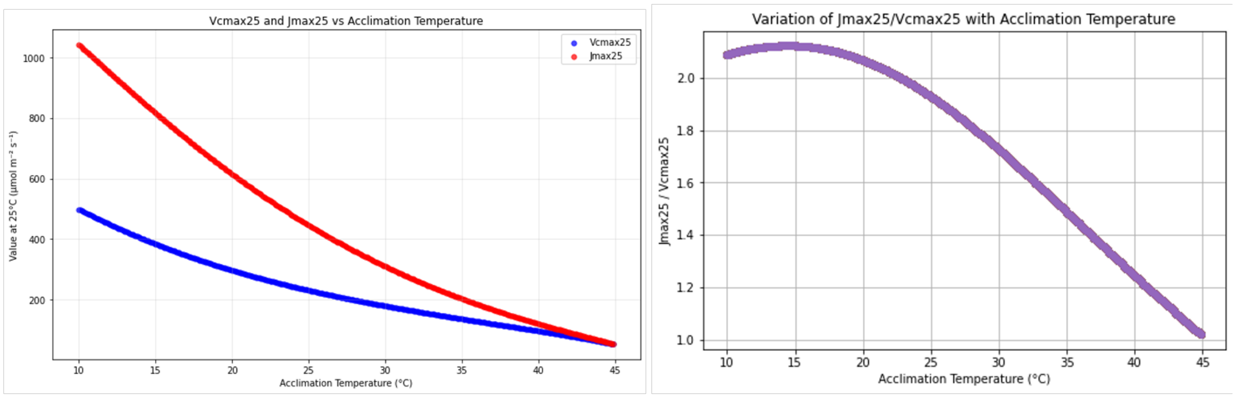

Fig. S5 Temperature response of J_{max} under different growth temperatures.

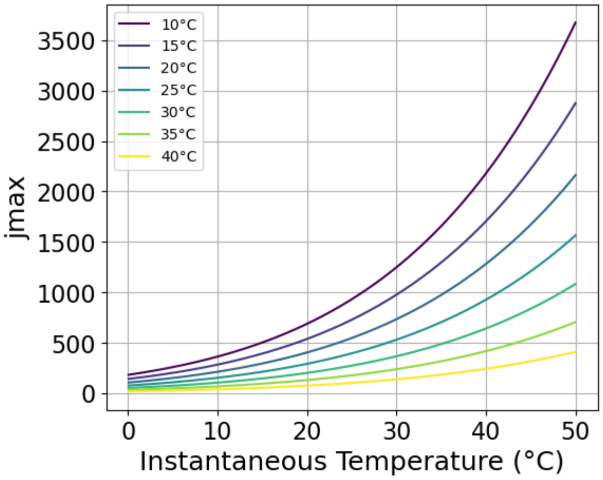

Fig. S6 Temperature responses of V_{cmax} and m_c. ($m_c = \frac{(C_i - \Gamma^*)}{(C_i + K)}$)

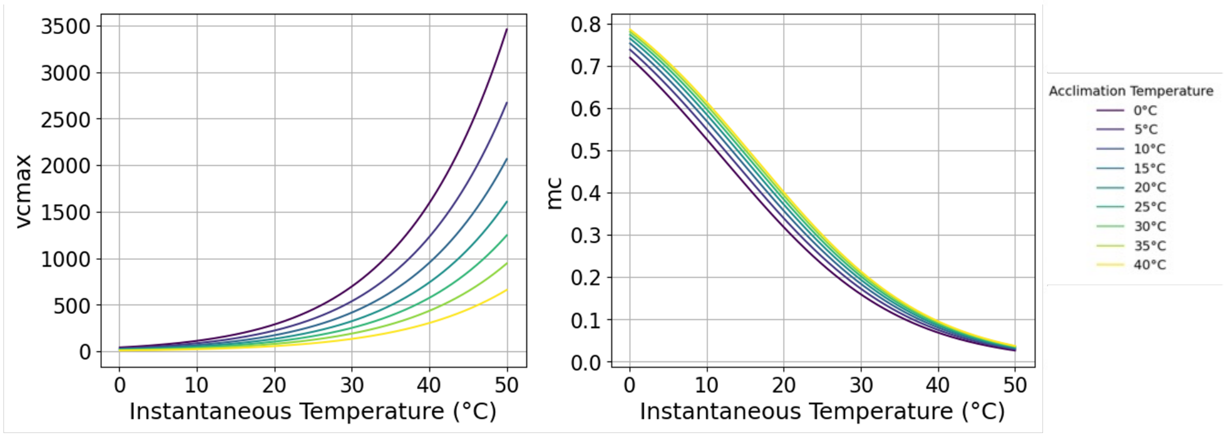

Fig. S7 T_{opt} and normalised A_{opt} versus growth temperature across all sites and simulation years.

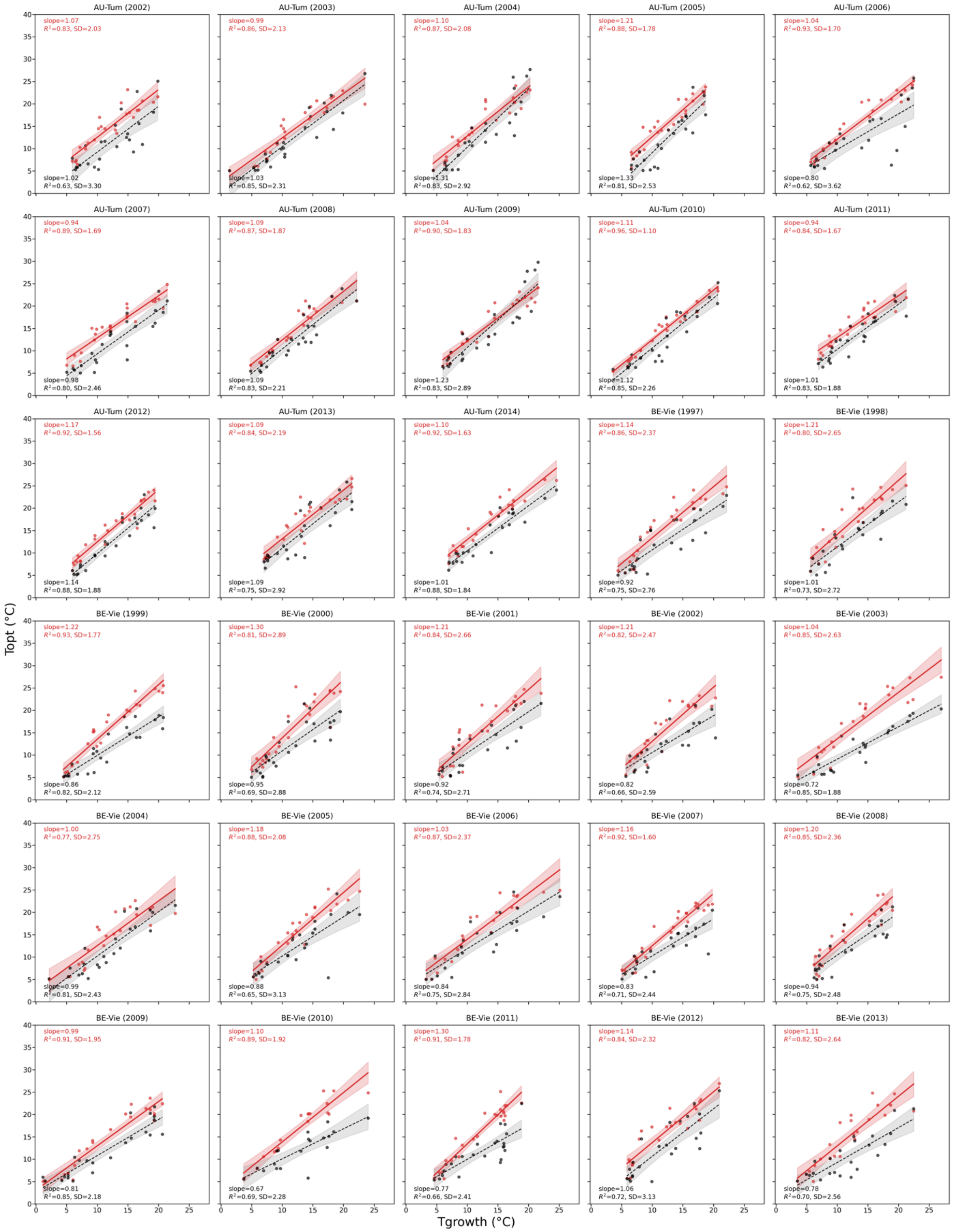

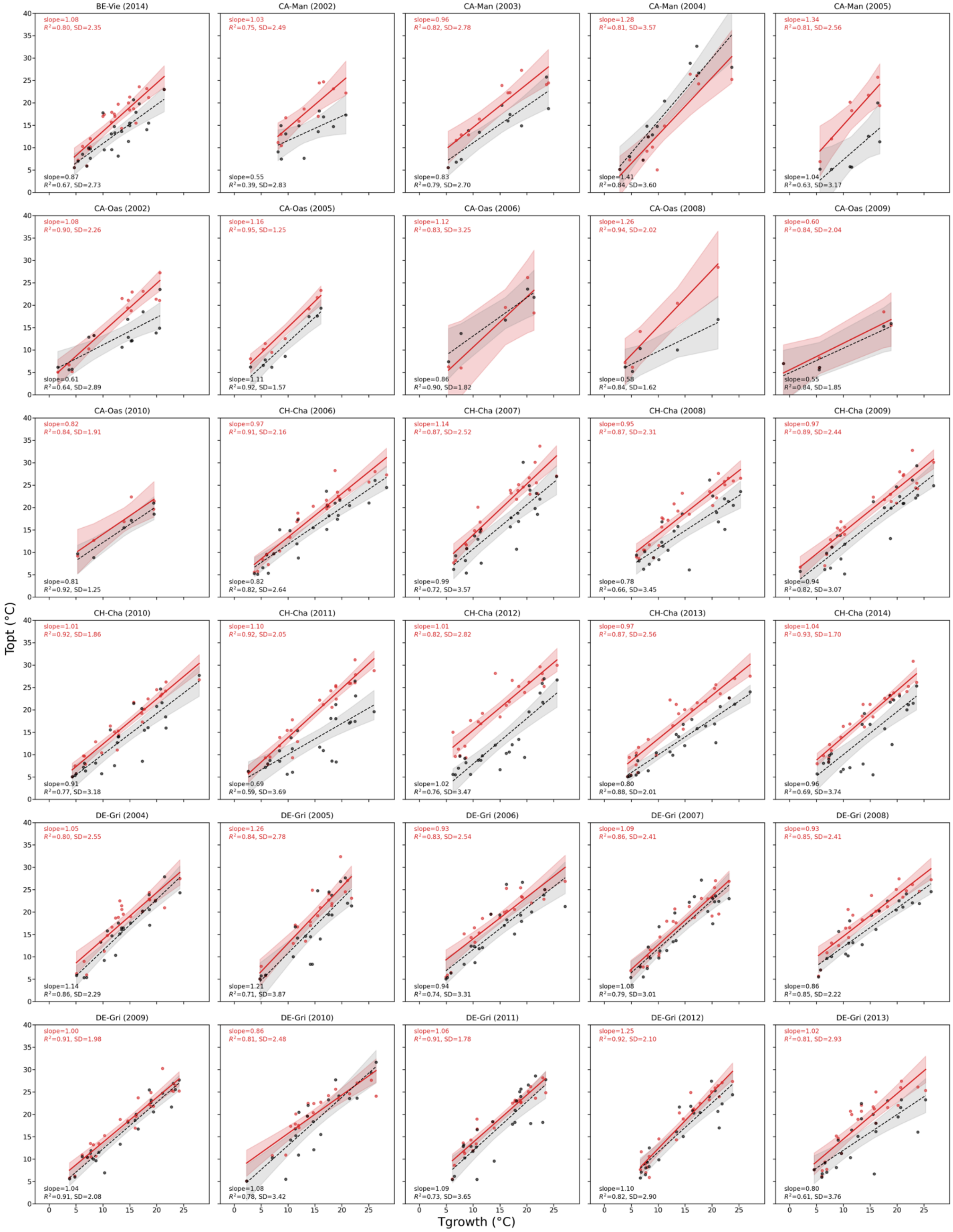

Topt (°C)
Tgrowth (°C)
BE-Vie (2014)
slope=1.08
R^2=0.80, SD=2.35
slope=0.87
R^2=0.67, SD=2.73
CA-Man (2002)
slope=1.03
R^2=0.75, SD=2.49
slope=0.55
R^2=0.39, SD=2.83
CA-Man (2003)
slope=0.96
R^2=0.82, SD=2.78
slope=0.83
R^2=0.79, SD=2.70
CA-Man (2004)
slope=1.28
R^2=0.81, SD=3.57
slope=1.41
R^2=0.84, SD=3.60
CA-Man (2005)
slope=1.34
R^2=0.81, SD=2.56
slope=1.04
R^2=0.63, SD=3.17
CA-Oas (2002)
slope=1.08
R^2=0.90, SD=2.26
slope=0.61
R^2=0.64, SD=2.89
CA-Oas (2005)
slope=1.16
R^2=0.95, SD=1.25
slope=1.11
R^2=0.92, SD=1.57
CA-Oas (2006)
slope=1.12
R^2=0.83, SD=3.25
slope=0.86
R^2=0.90, SD=1.82
CA-Oas (2008)
slope=1.26
R^2=0.94, SD=2.02
slope=0.58
R^2=0.84, SD=1.62
CA-Oas (2009)
slope=0.60
R^2=0.84, SD=2.04
slope=0.55
R^2=0.84, SD=1.85
CA-Oas (2010)
slope=0.82
R^2=0.84, SD=1.91
slope=0.81
R^2=0.92, SD=1.25
CH-Cha (2006)
slope=0.97
R^2=0.91, SD=2.16
slope=0.82
R^2=0.82, SD=2.64
CH-Cha (2007)
slope=1.14
R^2=0.87, SD=2.52
slope=0.99
R^2=0.72, SD=3.57
CH-Cha (2008)
slope=0.95
R^2=0.87, SD=2.31
slope=0.78
R^2=0.66, SD=3.45
CH-Cha (2009)
slope=0.97
R^2=0.89, SD=2.44
slope=0.94
R^2=0.82, SD=3.07
CH-Cha (2010)
slope=1.01
R^2=0.92, SD=1.86
slope=0.91
R^2=0.77, SD=3.18
CH-Cha (2011)
slope=1.10
R^2=0.92, SD=2.05
slope=0.69
R^2=0.59, SD=3.69
CH-Cha (2012)
slope=1.01
R^2=0.82, SD=2.82
slope=1.02
R^2=0.76, SD=3.47
CH-Cha (2013)
slope=0.97
R^2=0.87, SD=2.56
slope=0.80
R^2=0.88, SD=2.01
CH-Cha (2014)
slope=1.04
R^2=0.93, SD=1.70
slope=0.96
R^2=0.69, SD=3.74
DE-Gri (2004)
slope=1.05
R^2=0.80, SD=2.55
slope=1.14
R^2=0.86, SD=2.29
DE-Gri (2005)
slope=1.26
R^2=0.84, SD=2.78
slope=1.21
R^2=0.71, SD=3.87
DE-Gri (2006)
slope=0.93
R^2=0.83, SD=2.54
slope=0.94
R^2=0.74, SD=3.31
DE-Gri (2007)
slope=1.09
R^2=0.86, SD=2.41
slope=1.08
R^2=0.79, SD=3.01
DE-Gri (2008)
slope=0.93
R^2=0.85, SD=2.41
slope=0.86
R^2=0.85, SD=2.22
DE-Gri (2009)
slope=1.00
R^2=0.91, SD=1.98
slope=1.04
R^2=0.91, SD=2.08
DE-Gri (2010)
slope=0.86
R^2=0.81, SD=2.48
slope=1.08
R^2=0.78, SD=3.42
DE-Gri (2011)
slope=1.06
R^2=0.91, SD=1.78
slope=1.09
R^2=0.73, SD=3.65
DE-Gri (2012)
slope=1.25
R^2=0.92, SD=2.10
slope=1.10
R^2=0.82, SD=2.90
DE-Gri (2013)
slope=1.02
R^2=0.81, SD=2.93
slope=0.80
R^2=0.61, SD=3.76

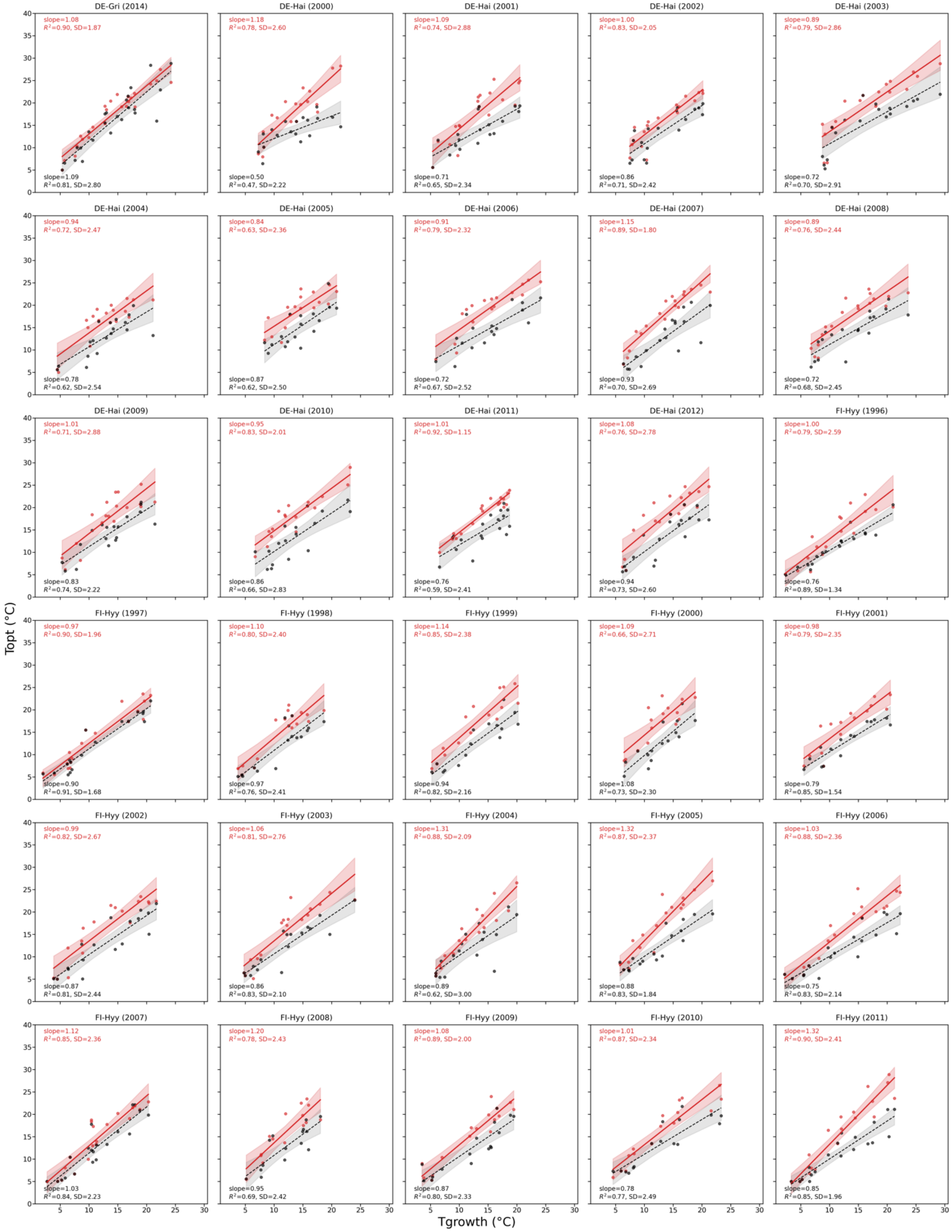

Topt (°C)
Tgrowth (°C)
DE-Gri (2014)
slope=1.08
R^2=0.90, SD=1.87
slope=1.09
R^2=0.81, SD=2.80
DE-Hai (2000)
slope=1.18
R^2=0.78, SD=2.60
slope=0.50
R^2=0.47, SD=2.22
DE-Hai (2001)
slope=1.09
R^2=0.74, SD=2.88
slope=0.71
R^2=0.65, SD=2.34
DE-Hai (2002)
slope=1.00
R^2=0.83, SD=2.05
slope=0.86
R^2=0.71, SD=2.42
DE-Hai (2003)
slope=0.89
R^2=0.79, SD=2.86
slope=0.72
R^2=0.70, SD=2.91
DE-Hai (2004)
slope=0.94
R^2=0.72, SD=2.47
slope=0.78
R^2=0.62, SD=2.54
DE-Hai (2005)
slope=0.84
R^2=0.63, SD=2.36
slope=0.87
R^2=0.62, SD=2.50
DE-Hai (2006)
slope=0.91
R^2=0.79, SD=2.32
slope=0.72
R^2=0.67, SD=2.52
DE-Hai (2007)
slope=1.15
R^2=0.89, SD=1.80
slope=0.93
R^2=0.70, SD=2.69
DE-Hai (2008)
slope=0.89
R^2=0.76, SD=2.44
slope=0.72
R^2=0.68, SD=2.45
DE-Hai (2009)
slope=1.01
R^2=0.71, SD=2.88
slope=0.83
R^2=0.74, SD=2.22
DE-Hai (2010)
slope=0.95
R^2=0.83, SD=2.01
slope=0.86
R^2=0.66, SD=2.83
DE-Hai (2011)
slope=1.01
R^2=0.92, SD=1.15
slope=0.76
R^2=0.59, SD=2.41
DE-Hai (2012)
slope=1.08
R^2=0.76, SD=2.78
slope=0.94
R^2=0.73, SD=2.60
FI-Hyy (1996)
slope=1.00
R^2=0.79, SD=2.59
slope=0.76
R^2=0.89, SD=1.34
FI-Hyy (1997)
slope=0.97
R^2=0.90, SD=1.96
slope=0.90
R^2=0.91, SD=1.68
FI-Hyy (1998)
slope=1.10
R^2=0.80, SD=2.40
slope=0.97
R^2=0.76, SD=2.41
FI-Hyy (1999)
slope=1.14
R^2=0.85, SD=2.38
slope=0.94
R^2=0.82, SD=2.16
FI-Hyy (2000)
slope=1.09
R^2=0.66, SD=2.71
slope=1.08
R^2=0.73, SD=2.30
FI-Hyy (2001)
slope=0.98
R^2=0.79, SD=2.35
slope=0.79
R^2=0.85, SD=1.54
FI-Hyy (2002)
slope=0.99
R^2=0.82, SD=2.67
slope=0.87
R^2=0.81, SD=2.44
FI-Hyy (2003)
slope=1.06
R^2=0.81, SD=2.76
slope=0.86
R^2=0.83, SD=2.10
FI-Hyy (2004)
slope=1.31
R^2=0.88, SD=2.09
slope=0.89
R^2=0.62, SD=3.00
FI-Hyy (2005)
slope=1.32
R^2=0.87, SD=2.37
slope=0.88
R^2=0.83, SD=1.84
FI-Hyy (2006)
slope=1.03
R^2=0.88, SD=2.36
slope=0.75
R^2=0.83, SD=2.14
FI-Hyy (2007)
slope=1.12
R^2=0.85, SD=2.36
slope=1.03
R^2=0.84, SD=2.23
FI-Hyy (2008)
slope=1.20
R^2=0.78, SD=2.43
slope=0.95
R^2=0.69, SD=2.42
FI-Hyy (2009)
slope=1.08
R^2=0.89, SD=2.00
slope=0.87
R^2=0.80, SD=2.33
FI-Hyy (2010)
slope=1.01
R^2=0.87, SD=2.34
slope=0.78
R^2=0.77, SD=2.49
FI-Hyy (2011)
slope=1.32
R^2=0.90, SD=2.41
slope=0.85
R^2=0.85, SD=1.96

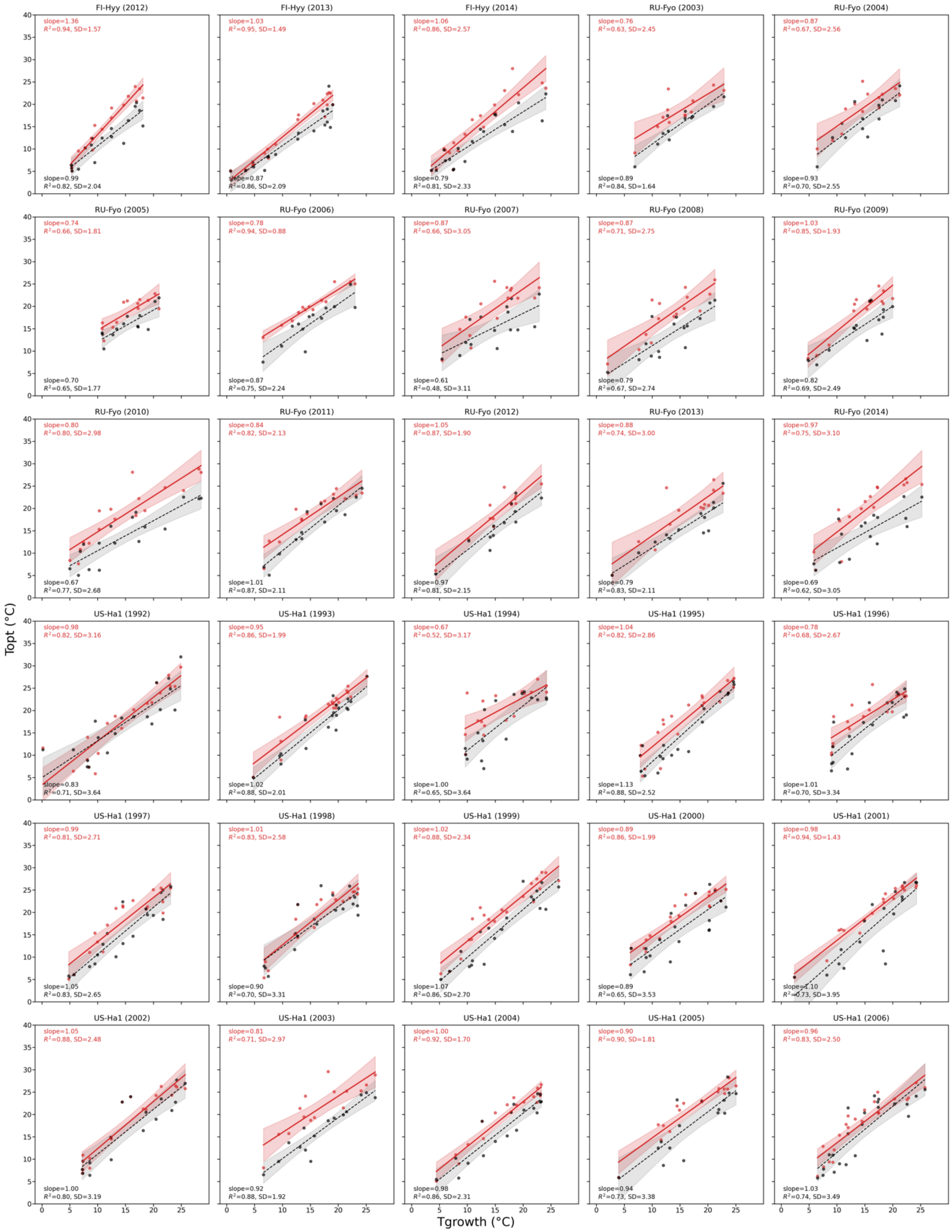

Topt (°C)
Tgrowth (°C)
FI-Hyy (2012)
slope=1.36
R^2=0.94, SD=1.57
slope=0.99
R^2=0.82, SD=2.04
FI-Hyy (2013)
slope=1.03
R^2=0.95, SD=1.49
slope=0.87
R^2=0.86, SD=2.09
FI-Hyy (2014)
slope=1.06
R^2=0.86, SD=2.57
slope=0.79
R^2=0.81, SD=2.33
RU-Fyo (2003)
slope=0.76
R^2=0.63, SD=2.45
slope=0.89
R^2=0.84, SD=1.64
RU-Fyo (2004)
slope=0.87
R^2=0.67, SD=2.56
slope=0.93
R^2=0.70, SD=2.55
RU-Fyo (2005)
slope=0.74
R^2=0.66, SD=1.81
slope=0.70
R^2=0.65, SD=1.77
RU-Fyo (2006)
slope=0.78
R^2=0.94, SD=0.88
slope=0.87
R^2=0.75, SD=2.24
RU-Fyo (2007)
slope=0.87
R^2=0.66, SD=3.05
slope=0.61
R^2=0.48, SD=3.11
RU-Fyo (2008)
slope=0.87
R^2=0.71, SD=2.75
slope=0.79
R^2=0.67, SD=2.74
RU-Fyo (2009)
slope=1.03
R^2=0.85, SD=1.93
slope=0.82
R^2=0.69, SD=2.49
RU-Fyo (2010)
slope=0.80
R^2=0.80, SD=2.98
slope=0.67
R^2=0.77, SD=2.68
RU-Fyo (2011)
slope=0.84
R^2=0.82, SD=2.13
slope=1.01
R^2=0.87, SD=2.11
RU-Fyo (2012)
slope=1.05
R^2=0.87, SD=1.90
slope=0.97
R^2=0.81, SD=2.15
RU-Fyo (2013)
slope=0.88
R^2=0.74, SD=3.00
slope=0.79
R^2=0.83, SD=2.11
RU-Fyo (2014)
slope=0.97
R^2=0.75, SD=3.10
slope=0.69
R^2=0.62, SD=3.05
US-Ha1 (1992)
slope=0.98
R^2=0.82, SD=3.16
slope=0.83
R^2=0.71, SD=3.64
US-Ha1 (1993)
slope=0.95
R^2=0.86, SD=1.99
slope=1.02
R^2=0.88, SD=2.01
US-Ha1 (1994)
slope=0.67
R^2=0.52, SD=3.17
slope=1.00
R^2=0.65, SD=3.64
US-Ha1 (1995)
slope=1.04
R^2=0.82, SD=2.86
slope=1.13
R^2=0.88, SD=2.52
US-Ha1 (1996)
slope=0.78
R^2=0.68, SD=2.67
slope=1.01
R^2=0.70, SD=3.34
US-Ha1 (1997)
slope=0.99
R^2=0.81, SD=2.71
slope=1.05
R^2=0.83, SD=2.65
US-Ha1 (1998)
slope=1.01
R^2=0.83, SD=2.58
slope=0.90
R^2=0.70, SD=3.31
US-Ha1 (1999)
slope=1.02
R^2=0.88, SD=2.34
slope=1.07
R^2=0.86, SD=2.70
US-Ha1 (2000)
slope=0.89
R^2=0.86, SD=1.99
slope=0.89
R^2=0.65, SD=3.53
US-Ha1 (2001)
slope=0.98
R^2=0.94, SD=1.43
slope=1.10
R^2=0.73, SD=3.95
US-Ha1 (2002)
slope=1.05
R^2=0.88, SD=2.48
slope=1.00
R^2=0.80, SD=3.19
US-Ha1 (2003)
slope=0.81
R^2=0.71, SD=2.97
slope=0.92
R^2=0.88, SD=1.92
US-Ha1 (2004)
slope=1.00
R^2=0.92, SD=1.70
slope=0.98
R^2=0.86, SD=2.31
US-Ha1 (2005)
slope=0.90
R^2=0.90, SD=1.81
slope=0.94
R^2=0.73, SD=3.38
US-Ha1 (2006)
slope=0.96
R^2=0.83, SD=2.50
slope=1.03
R^2=0.74, SD=3.49

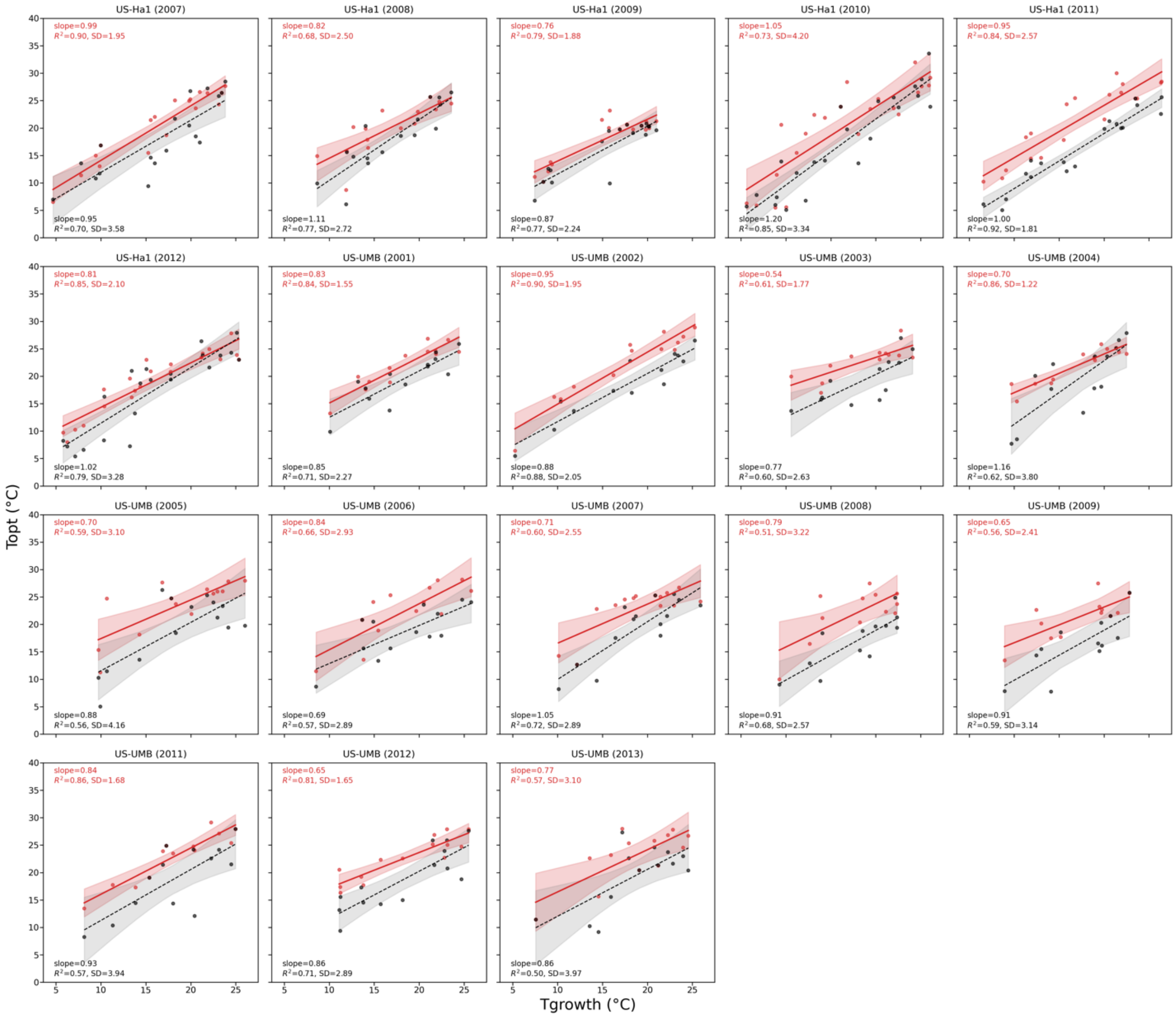

US-Ha1 (2007)
slope=0.99
R^2=0.90, SD=1.95
slope=0.95
R^2=0.70, SD=3.58
US-Ha1 (2008)
slope=0.82
R^2=0.68, SD=2.50
slope=1.11
R^2=0.77, SD=2.72
US-Ha1 (2009)
slope=0.76
R^2=0.79, SD=1.88
slope=0.87
R^2=0.77, SD=2.24
US-Ha1 (2010)
slope=1.05
R^2=0.73, SD=4.20
slope=1.20
R^2=0.85, SD=3.34
US-Ha1 (2011)
slope=0.95
R^2=0.84, SD=2.57
slope=1.00
R^2=0.92, SD=1.81
US-Ha1 (2012)
slope=0.81
R^2=0.85, SD=2.10
slope=1.02
R^2=0.79, SD=3.28
US-UMB (2001)
slope=0.83
R^2=0.84, SD=1.55
slope=0.85
R^2=0.71, SD=2.27
US-UMB (2002)
slope=0.95
R^2=0.90, SD=1.95
slope=0.88
R^2=0.88, SD=2.05
US-UMB (2003)
slope=0.54
R^2=0.61, SD=1.77
slope=0.77
R^2=0.60, SD=2.63
US-UMB (2004)
slope=0.70
R^2=0.86, SD=1.22
slope=1.16
R^2=0.62, SD=3.80
US-UMB (2005)
slope=0.70
R^2=0.59, SD=3.10
slope=0.88
R^2=0.56, SD=4.16
US-UMB (2006)
slope=0.84
R^2=0.66, SD=2.93
slope=0.69
R^2=0.57, SD=2.89
US-UMB (2007)
slope=0.71
R^2=0.60, SD=2.55
slope=1.05
R^2=0.72, SD=2.89
US-UMB (2008)
slope=0.79
R^2=0.51, SD=3.22
slope=0.91
R^2=0.68, SD=2.57
US-UMB (2009)
slope=0.65
R^2=0.56, SD=2.41
slope=0.91
R^2=0.59, SD=3.14
US-UMB (2011)
slope=0.84
R^2=0.86, SD=1.68
slope=0.93
R^2=0.57, SD=3.94
US-UMB (2012)
slope=0.65
R^2=0.81, SD=1.65
slope=0.86
R^2=0.71, SD=2.89
US-UMB (2013)
slope=0.77
R^2=0.57, SD=3.10
slope=0.86
R^2=0.50, SD=3.97
Topt (°C)
Tgrowth (°C)

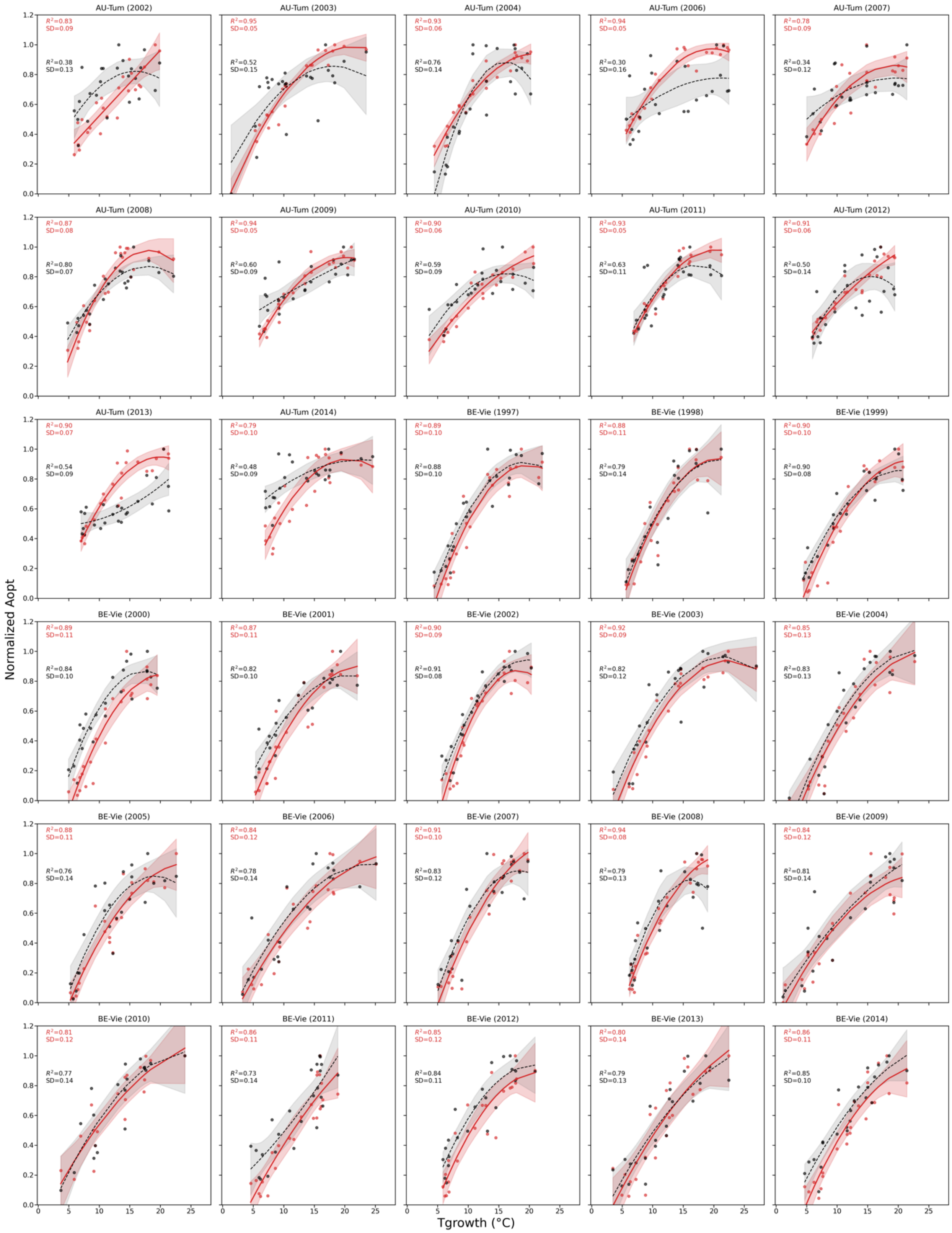
AU-Tum (2002)
R^2=0.83 SD=0.09
R^2=0.38 SD=0.13
AU-Tum (2003)
R^2=0.95 SD=0.05
R^2=0.52 SD=0.15
AU-Tum (2004)
R^2=0.93 SD=0.06
R^2=0.76 SD=0.14
AU-Tum (2006)
R^2=0.94 SD=0.05
R^2=0.30 SD=0.16
AU-Tum (2007)
R^2=0.78 SD=0.09
R^2=0.34 SD=0.12
AU-Tum (2008)
R^2=0.87 SD=0.08
R^2=0.80 SD=0.07
AU-Tum (2009)
R^2=0.94 SD=0.05
R^2=0.60 SD=0.09
AU-Tum (2010)
R^2=0.90 SD=0.06
R^2=0.59 SD=0.09
AU-Tum (2011)
R^2=0.93 SD=0.05
R^2=0.63 SD=0.11
AU-Tum (2012)
R^2=0.91 SD=0.06
R^2=0.50 SD=0.14
AU-Tum (2013)
R^2=0.90 SD=0.07
R^2=0.54 SD=0.09
AU-Tum (2014)
R^2=0.79 SD=0.10
R^2=0.48 SD=0.09
BE-Vie (1997)
R^2=0.89 SD=0.10
R^2=0.88 SD=0.10
BE-Vie (1998)
R^2=0.88 SD=0.11
R^2=0.79 SD=0.14
BE-Vie (1999)
R^2=0.90 SD=0.10
R^2=0.90 SD=0.08
BE-Vie (2000)
R^2=0.89 SD=0.11
R^2=0.84 SD=0.10
BE-Vie (2001)
R^2=0.87 SD=0.11
R^2=0.82 SD=0.10
BE-Vie (2002)
R^2=0.90 SD=0.09
R^2=0.91 SD=0.08
BE-Vie (2003)
R^2=0.92 SD=0.09
R^2=0.82 SD=0.12
BE-Vie (2004)
R^2=0.85 SD=0.13
R^2=0.83 SD=0.13
BE-Vie (2005)
R^2=0.88 SD=0.11
R^2=0.76 SD=0.14
BE-Vie (2006)
R^2=0.84 SD=0.12
R^2=0.78 SD=0.14
BE-Vie (2007)
R^2=0.91 SD=0.10
R^2=0.83 SD=0.12
BE-Vie (2008)
R^2=0.94 SD=0.08
R^2=0.79 SD=0.13
BE-Vie (2009)
R^2=0.84 SD=0.12
R^2=0.81 SD=0.14
BE-Vie (2010)
R^2=0.81 SD=0.12
R^2=0.77 SD=0.14
BE-Vie (2011)
R^2=0.86 SD=0.11
R^2=0.73 SD=0.14
BE-Vie (2012)
R^2=0.85 SD=0.12
R^2=0.84 SD=0.11
BE-Vie (2013)
R^2=0.80 SD=0.14
R^2=0.79 SD=0.13
BE-Vie (2014)
R^2=0.86 SD=0.11
R^2=0.85 SD=0.10
Normalized Aopt
Tgrowth (°C)

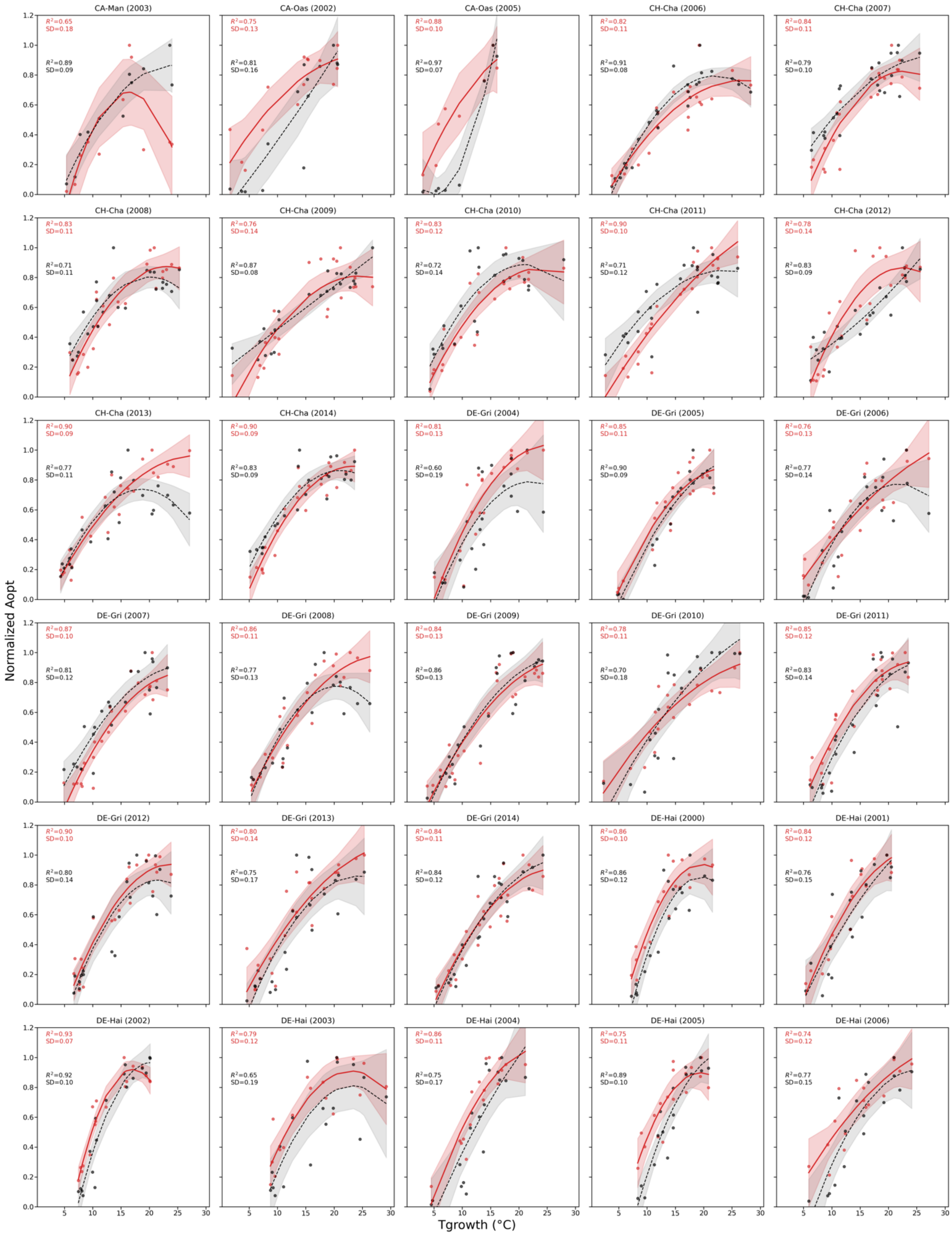
CA-Man (2003)
R²=0.65 SD=0.18
R²=0.89 SD=0.09
CA-Oas (2002)
R²=0.75 SD=0.13
R²=0.81 SD=0.16
CA-Oas (2005)
R²=0.88 SD=0.10
R²=0.97 SD=0.07
CH-Cha (2006)
R²=0.82 SD=0.11
R²=0.91 SD=0.08
CH-Cha (2007)
R²=0.84 SD=0.11
R²=0.79 SD=0.10
CH-Cha (2008)
R²=0.83 SD=0.11
R²=0.71 SD=0.11
CH-Cha (2009)
R²=0.76 SD=0.14
R²=0.87 SD=0.08
CH-Cha (2010)
R²=0.83 SD=0.12
R²=0.72 SD=0.14
CH-Cha (2011)
R²=0.90 SD=0.10
R²=0.71 SD=0.12
CH-Cha (2012)
R²=0.78 SD=0.14
R²=0.83 SD=0.09
CH-Cha (2013)
R²=0.90 SD=0.09
R²=0.77 SD=0.11
CH-Cha (2014)
R²=0.90 SD=0.09
R²=0.83 SD=0.09
DE-Gri (2004)
R²=0.81 SD=0.13
R²=0.60 SD=0.19
DE-Gri (2005)
R²=0.85 SD=0.11
R²=0.90 SD=0.09
DE-Gri (2006)
R²=0.76 SD=0.13
R²=0.77 SD=0.14
DE-Gri (2007)
R²=0.87 SD=0.10
R²=0.81 SD=0.12
DE-Gri (2008)
R²=0.86 SD=0.11
R²=0.77 SD=0.13
DE-Gri (2009)
R²=0.84 SD=0.13
R²=0.86 SD=0.13
DE-Gri (2010)
R²=0.78 SD=0.11
R²=0.70 SD=0.18
DE-Gri (2011)
R²=0.85 SD=0.12
R²=0.83 SD=0.14
DE-Gri (2012)
R²=0.90 SD=0.10
R²=0.80 SD=0.14
DE-Gri (2013)
R²=0.80 SD=0.14
R²=0.75 SD=0.17
DE-Gri (2014)
R²=0.84 SD=0.11
R²=0.84 SD=0.12
DE-Hai (2000)
R²=0.86 SD=0.10
R²=0.86 SD=0.12
DE-Hai (2001)
R²=0.84 SD=0.12
R²=0.76 SD=0.15
DE-Hai (2002)
R²=0.93 SD=0.07
R²=0.92 SD=0.10
DE-Hai (2003)
R²=0.79 SD=0.12
R²=0.65 SD=0.19
DE-Hai (2004)
R²=0.86 SD=0.11
R²=0.75 SD=0.17
DE-Hai (2005)
R²=0.75 SD=0.11
R²=0.89 SD=0.10
DE-Hai (2006)
R²=0.74 SD=0.12
R²=0.77 SD=0.15
Normalized Aopt
Tgrowth (°C)

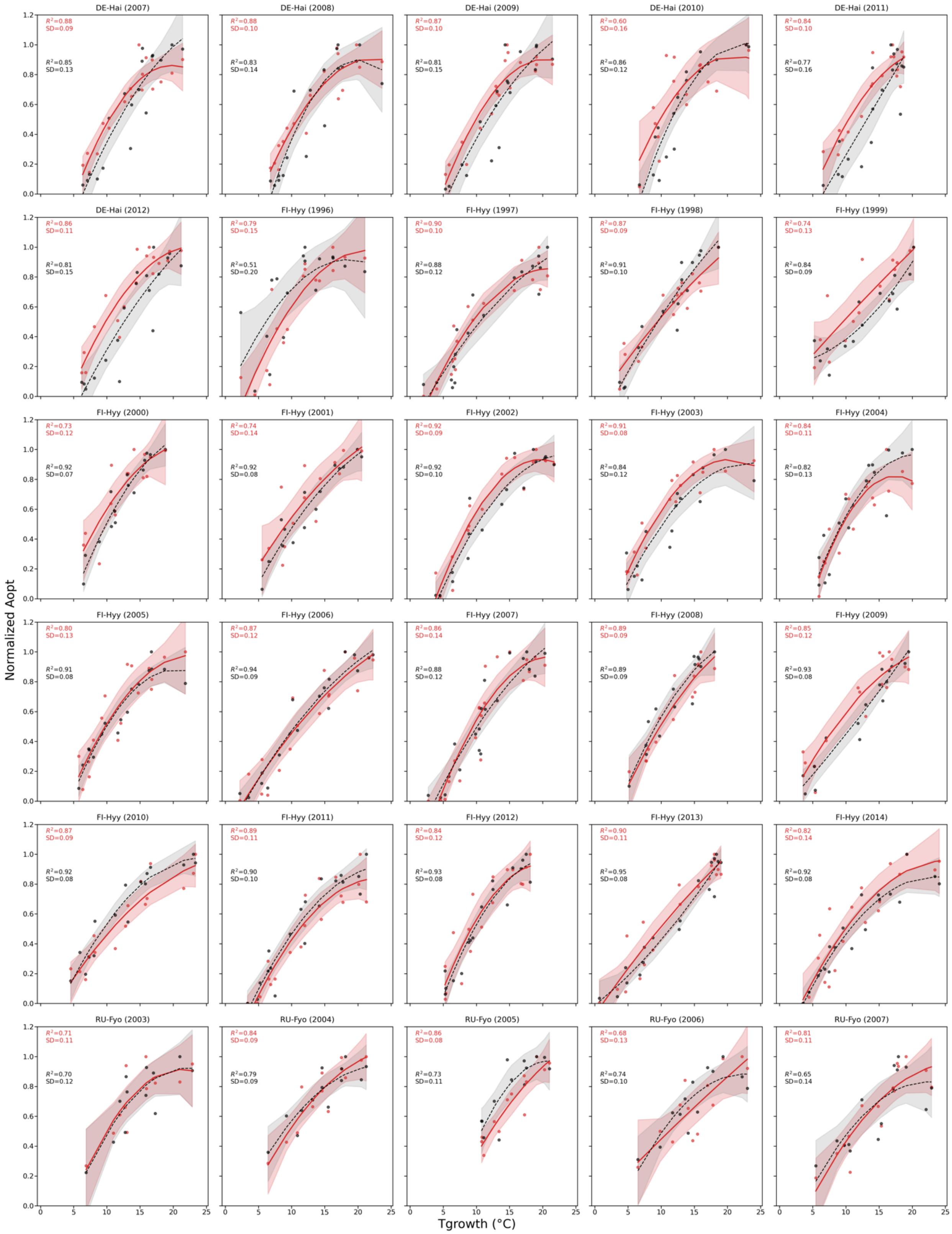
DE-Hai (2007)
R^2=0.88 SD=0.09
R^2=0.85 SD=0.13
DE-Hai (2008)
R^2=0.88 SD=0.10
R^2=0.83 SD=0.14
DE-Hai (2009)
R^2=0.87 SD=0.10
R^2=0.81 SD=0.15
DE-Hai (2010)
R^2=0.60 SD=0.16
R^2=0.86 SD=0.12
DE-Hai (2011)
R^2=0.84 SD=0.10
R^2=0.77 SD=0.16
DE-Hai (2012)
R^2=0.86 SD=0.11
R^2=0.81 SD=0.15
FI-Hyy (1996)
R^2=0.79 SD=0.15
R^2=0.51 SD=0.20
FI-Hyy (1997)
R^2=0.90 SD=0.10
R^2=0.88 SD=0.12
FI-Hyy (1998)
R^2=0.87 SD=0.09
R^2=0.91 SD=0.10
FI-Hyy (1999)
R^2=0.74 SD=0.13
R^2=0.84 SD=0.09
FI-Hyy (2000)
R^2=0.73 SD=0.12
R^2=0.92 SD=0.07
FI-Hyy (2001)
R^2=0.74 SD=0.14
R^2=0.92 SD=0.08
FI-Hyy (2002)
R^2=0.92 SD=0.09
R^2=0.92 SD=0.10
FI-Hyy (2003)
R^2=0.91 SD=0.08
R^2=0.84 SD=0.12
FI-Hyy (2004)
R^2=0.84 SD=0.11
R^2=0.82 SD=0.13
FI-Hyy (2005)
R^2=0.80 SD=0.13
R^2=0.91 SD=0.08
FI-Hyy (2006)
R^2=0.87 SD=0.12
R^2=0.94 SD=0.09
FI-Hyy (2007)
R^2=0.86 SD=0.14
R^2=0.88 SD=0.12
FI-Hyy (2008)
R^2=0.89 SD=0.09
R^2=0.89 SD=0.09
FI-Hyy (2009)
R^2=0.85 SD=0.12
R^2=0.93 SD=0.08
FI-Hyy (2010)
R^2=0.87 SD=0.09
R^2=0.92 SD=0.08
FI-Hyy (2011)
R^2=0.89 SD=0.11
R^2=0.90 SD=0.10
FI-Hyy (2012)
R^2=0.84 SD=0.12
R^2=0.93 SD=0.08
FI-Hyy (2013)
R^2=0.90 SD=0.11
R^2=0.95 SD=0.08
FI-Hyy (2014)
R^2=0.82 SD=0.14
R^2=0.92 SD=0.08
RU-Fyo (2003)
R^2=0.71 SD=0.11
R^2=0.70 SD=0.12
RU-Fyo (2004)
R^2=0.84 SD=0.09
R^2=0.79 SD=0.09
RU-Fyo (2005)
R^2=0.86 SD=0.08
R^2=0.73 SD=0.11
RU-Fyo (2006)
R^2=0.68 SD=0.13
R^2=0.74 SD=0.10
RU-Fyo (2007)
R^2=0.81 SD=0.11
R^2=0.65 SD=0.14
Normalized Aopt
Tgrowth (°C)

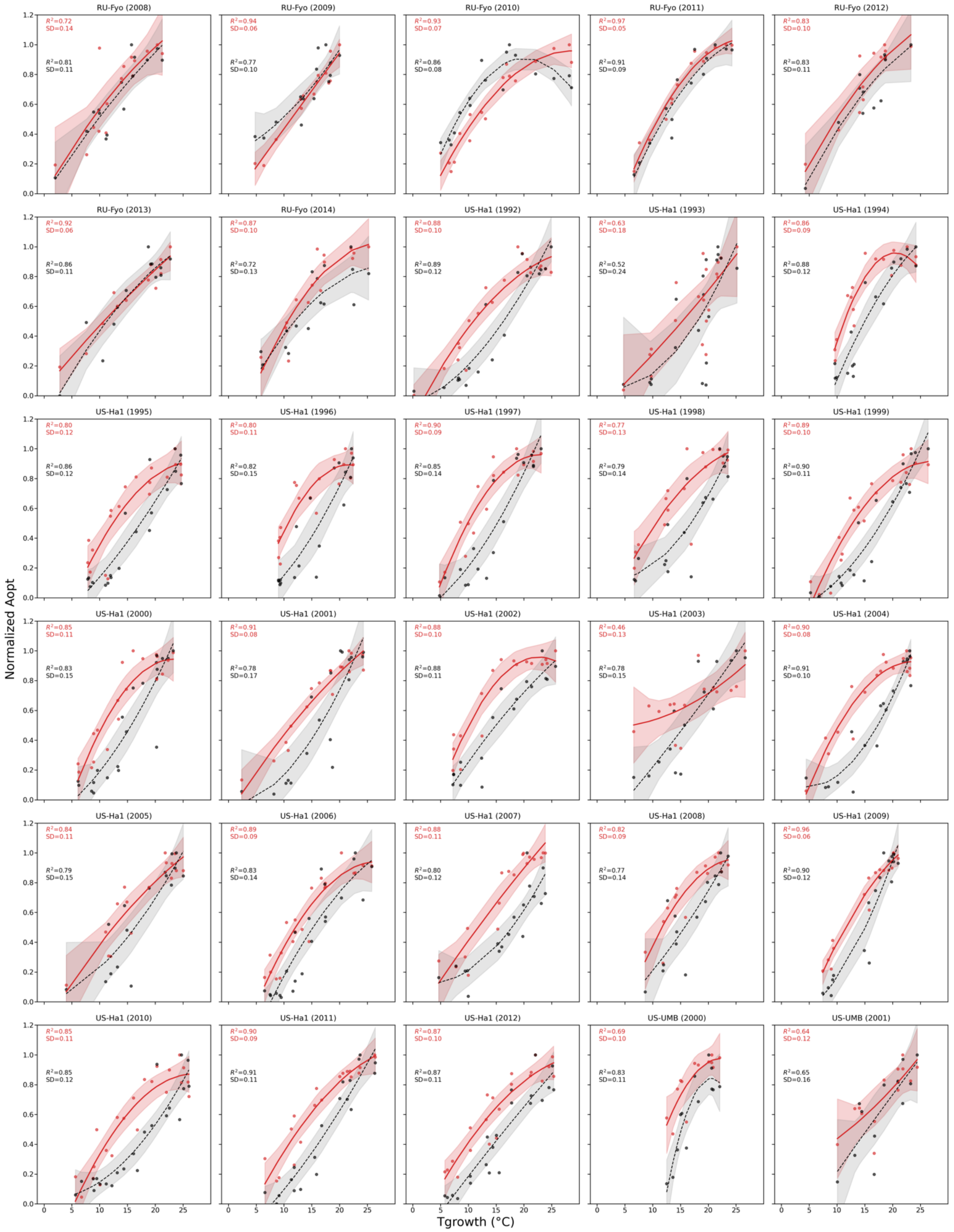

RU-Fyo (2008)
R^2=0.72 SD=0.14
R^2=0.81 SD=0.11
RU-Fyo (2009)
R^2=0.94 SD=0.06
R^2=0.77 SD=0.10
RU-Fyo (2010)
R^2=0.93 SD=0.07
R^2=0.86 SD=0.08
RU-Fyo (2011)
R^2=0.97 SD=0.05
R^2=0.91 SD=0.09
RU-Fyo (2012)
R^2=0.83 SD=0.10
R^2=0.83 SD=0.11
RU-Fyo (2013)
R^2=0.92 SD=0.06
R^2=0.86 SD=0.11
RU-Fyo (2014)
R^2=0.87 SD=0.10
R^2=0.72 SD=0.13
US-Ha1 (1992)
R^2=0.88 SD=0.10
R^2=0.89 SD=0.12
US-Ha1 (1993)
R^2=0.63 SD=0.18
R^2=0.52 SD=0.24
US-Ha1 (1994)
R^2=0.86 SD=0.09
R^2=0.88 SD=0.12
US-Ha1 (1995)
R^2=0.80 SD=0.12
R^2=0.86 SD=0.12
US-Ha1 (1996)
R^2=0.80 SD=0.11
R^2=0.82 SD=0.15
US-Ha1 (1997)
R^2=0.90 SD=0.09
R^2=0.85 SD=0.14
US-Ha1 (1998)
R^2=0.77 SD=0.13
R^2=0.79 SD=0.14
US-Ha1 (1999)
R^2=0.89 SD=0.10
R^2=0.90 SD=0.11
US-Ha1 (2000)
R^2=0.85 SD=0.11
R^2=0.83 SD=0.15
US-Ha1 (2001)
R^2=0.91 SD=0.08
R^2=0.78 SD=0.17
US-Ha1 (2002)
R^2=0.88 SD=0.10
R^2=0.88 SD=0.11
US-Ha1 (2003)
R^2=0.46 SD=0.13
R^2=0.78 SD=0.15
US-Ha1 (2004)
R^2=0.90 SD=0.08
R^2=0.91 SD=0.10
US-Ha1 (2005)
R^2=0.84 SD=0.11
R^2=0.79 SD=0.15
US-Ha1 (2006)
R^2=0.89 SD=0.09
R^2=0.83 SD=0.14
US-Ha1 (2007)
R^2=0.88 SD=0.11
R^2=0.80 SD=0.12
US-Ha1 (2008)
R^2=0.82 SD=0.09
R^2=0.77 SD=0.14
US-Ha1 (2009)
R^2=0.96 SD=0.06
R^2=0.90 SD=0.12
US-Ha1 (2010)
R^2=0.85 SD=0.11
R^2=0.85 SD=0.12
US-Ha1 (2011)
R^2=0.90 SD=0.09
R^2=0.91 SD=0.11
US-Ha1 (2012)
R^2=0.87 SD=0.10
R^2=0.87 SD=0.11
US-UMB (2000)
R^2=0.69 SD=0.10
R^2=0.83 SD=0.11
US-UMB (2001)
R^2=0.64 SD=0.12
R^2=0.65 SD=0.16
Normalized Aopt
Tgrowth (°C)

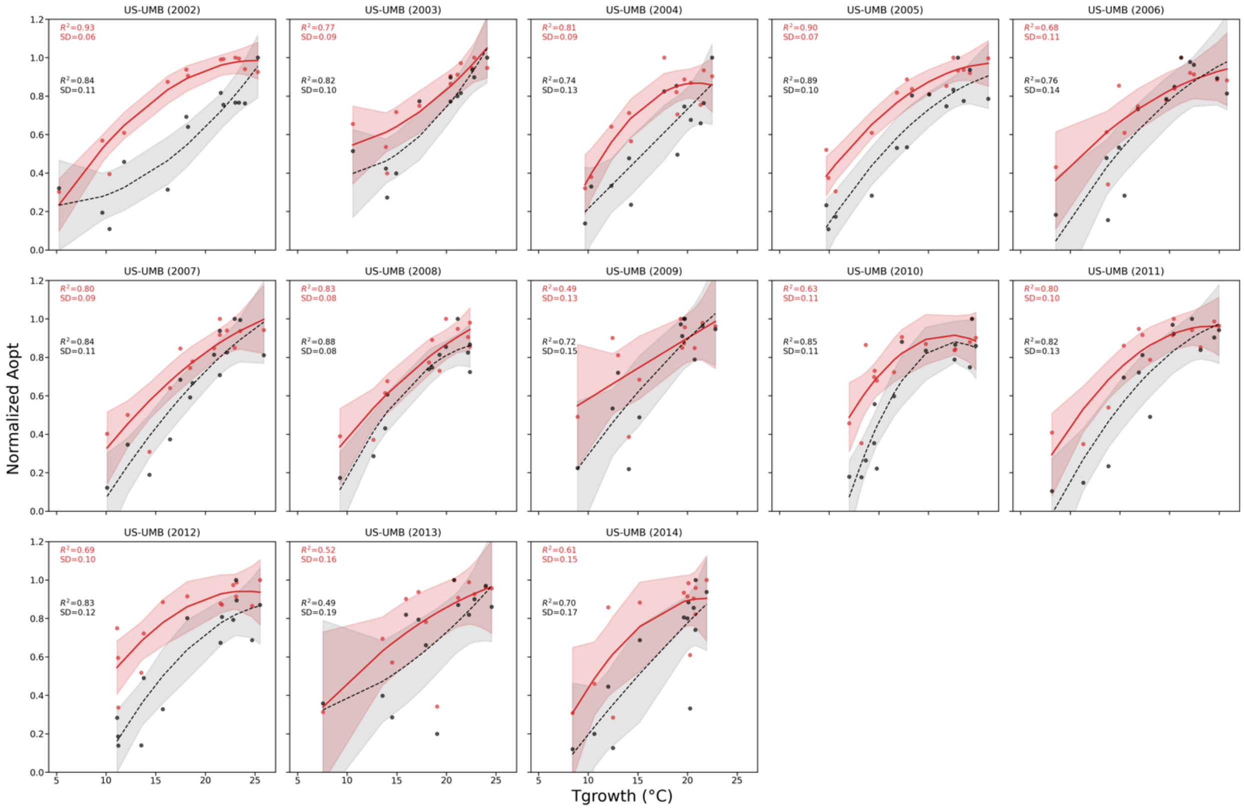

US-UMB (2002)
R^2=0.93
SD=0.06
R^2=0.84
SD=0.11
US-UMB (2003)
R^2=0.77
SD=0.09
R^2=0.82
SD=0.10
US-UMB (2004)
R^2=0.81
SD=0.09
R^2=0.74
SD=0.13
US-UMB (2005)
R^2=0.90
SD=0.07
R^2=0.89
SD=0.10
US-UMB (2006)
R^2=0.68
SD=0.11
R^2=0.76
SD=0.14
US-UMB (2007)
R^2=0.80
SD=0.09
R^2=0.84
SD=0.11
US-UMB (2008)
R^2=0.83
SD=0.08
R^2=0.88
SD=0.08
US-UMB (2009)
R^2=0.49
SD=0.13
R^2=0.72
SD=0.15
US-UMB (2010)
R^2=0.63
SD=0.11
R^2=0.85
SD=0.11
US-UMB (2011)
R^2=0.80
SD=0.10
R^2=0.82
SD=0.13
US-UMB (2012)
R^2=0.69
SD=0.10
R^2=0.83
SD=0.12
US-UMB (2013)
R^2=0.52
SD=0.16
R^2=0.49
SD=0.19
US-UMB (2014)
R^2=0.61
SD=0.15
R^2=0.70
SD=0.17
Normalized Aopt
Tgrowth (°C)

Fig. S8 Effects of leaf–air temperature differences (ΔT) on the apparent optimum temperature and GPP–temperature responses. (a) Changes in apparent optimum temperature (ΔTopt) with ΔT across growth air temperatures from 10 to 40 °C. Line colours indicate growth temperatures: navy = 10 °C, orange = 15 °C, green = 20 °C, red = 25 °C, purple = 30 °C, brown = 35 °C, and pink = 40 °C. (b) Instantaneous GPP–leaf temperature response curves under different ΔT values at a growth air temperature of 20 °C. Line colours indicate the ΔT settings: dark blue = −2 °C, black = 0 °C, orange = +2 °C, and dark red = +8 °C. All simulations used PAR = 1800 μmol m^{-2} s^{-1}, Patm = 101 325 Pa, CO_2 = 400 ppm, and a VPD that increased with temperature while vapour pressure was fixed at 500 Pa.

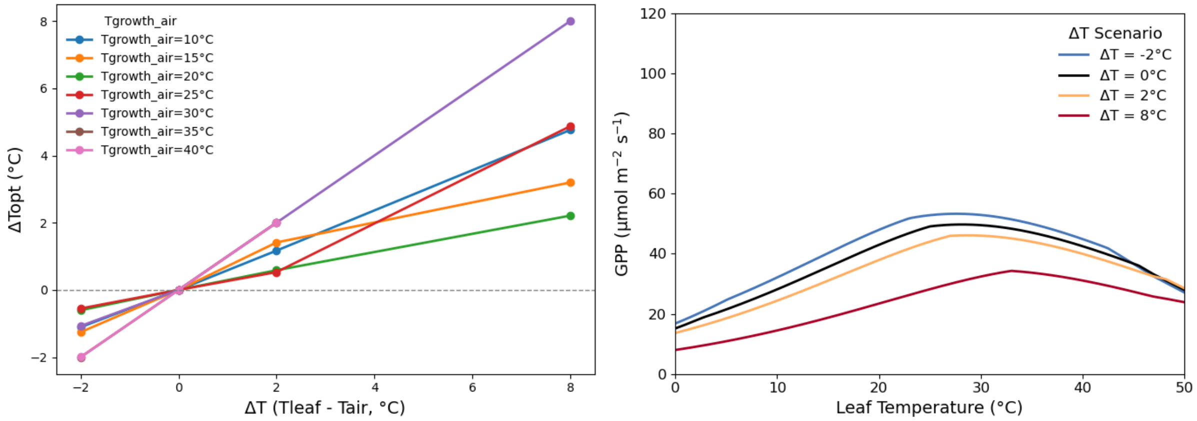

Table S1 Description of the gas exchange measurement dataset (Adapted from Kumarathunge et al., 2018).

Dataset	Species	Seed source location (latitude longitude)	Data collection periods	Data collection location	Age of plants	Data type	Instrument used	Reference
Eucalyptus tereticornis provs AU-NSW	*Eucalyptus tereticornis* prov. temperate prov. tropical	-35.39 150.07 -15.5 145.14	February 2016	Climate controlled glasshouse managed by the Western Sydney University, Australia	seedlings	ACi-T	Licor 6400	Crous et al. (2018)
Ghannoum Eucalypt spp, AU-NSW	*Eucalyptus saligna, Eucalyptus sideroxylon*	-30.57 152.15 -32.99 147.89		Climate controlled glasshouse managed by the Western Sydney University, Australia	seedlings	An-T	Licor 6400	Ghannoum et al. (2010)
Smith C3 spp, IN, USA	*Acer rubrum*	Exact seed source		Climate controlled growth	seedlings	ACi-T	Licor 6400	Smith and Dukes (2017)

Dataset	Species	Seed source location (latitude longitude)	Data collection periods	Data collection location	Age of plants	Data type	Instrument used	Reference
	Betula alleghaniensis<br>*Cedrela odorata*<br>*Elymus Canadensis*<br>*Glycine max*<br>*Pinus nigra*<br>*Pinus pinaster*<br>*Pinus pinea*<br>*Pinus sylvestris*<br>*Poa pratensis*<br>*Quercus virginiana*	locations were unknown.		chambers managed by the Purdue University				

Dataset	Species	Seed source location (latitude longitude)	Data collection periods	Data collection location	Age of plants	Data type	Instrument used	Reference
	Tamarindus indica							
	Triticum aestivum							
	Ulmus americana							

Table S2 Description of the FLUXNET dataset

SiteID	Climate	Simulation years	Reference
AU-Tum	Cfb	2002 - 2014	Leuning et al. (2005)
BE-Vie	Cfb	1997 - 2014	Aubinet et al. (2001)
CA-Man	Dfc	2002 - 2006	Dunn et al. (2007)
CA-Oas	Dfc	2002 - 2010	Black (2016)
CH-Cha	Cfb	2006 - 2014	Merbold et al. (2014)
DE-Gri	Cfb	2004 - 2014	Prescher et al. (2010)
DE-Hai	DBF	2000 - 2014	Knohl et al. (2003)
FI-Hyy	Dfc	1996 - 2012	Suni et al. (2003)
RU-Fyo	Dfb	2003 - 2014	Kurbatova et al. (2008)
US-Ha1	Dfb	1992 - 2012	Urbanskiet al. (2007)
US-UMB	Dfb	1999 - 2014	Gough et al. (2013)

Table S3 Summary of ΔT scenarios and corresponding changes in modelled photosynthetic optimum temperature (T_{opt_leaf}) across growth air temperatures (T_{growth_air}).

ΔT	T_{growth_air}	T_{growth_leaf}	T_{opt_leaf}	ΔT_{opt}
-2	10	8	20.2	-1.1
0	10	10	21.4	0.0
2	10	12	22.5	1.2
8	10	18	26.1	4.8
-2	15	13	23.1	-1.3
0	15	15	24.4	0.0
2	15	17	25.8	1.4
8	15	23	27.6	3.2
-2	20	18	26.1	-0.6
0	20	20	26.7	0.0
2	20	22	27.3	0.6
8	20	28	28.9	2.2
-2	25	23	27.6	-0.6
0	25	25	28.1	0.0
2	25	27	28.7	0.5
8	25	33	33.0	4.9
-2	30	28	28.9	-1.1
0	30	30	30.0	0.0
2	30	32	32.0	2.0
8	30	38	38.0	8.0
-2	35	33	33.0	-2.0
0	35	35	35.0	0.0
2	35	37	37.0	2.0
-2	40	38	38.0	-2.0
0	40	40	40.0	0.0

Notes S1. Interpretation of the coordination hypothesis as an optimality criterion

A simple derivation is as follows. According to the FvCB model, the gross assimilation rate (A) is given by:

$$A = \min(A_J, A_C)$$

where A_J is the electron-transport limited rate, given by

$$A_J = (J/4)\,(c_i - \Gamma^*)/(c_i + 2\Gamma^*)$$

with J being the electron transport rate, c_i the leaf-internal CO_2 partial pressure and Γ^* the photorespiratory compensation point; and A_C is the Rubisco-limited rate, given by

$$A_C = V_{cmax}\,(c_i - \Gamma^*)/(c_i + K)$$

with V_{cmax} being the maximum rate of carboxylation and K being the effective Michaelis-Menten coefficient of Rubisco. The net assimilation rate is:

$$A_n = A - R_d$$

where R_d is the leaf respiration rate. In addition,

$$R_d = b\,V_{cmax}$$

where b is a small constant, often taken to be 0.015 at 25˚C.

Consider first the case where $A_J > A_C$. Then A_n is given by $V_{cmax}\,[(c_i - \Gamma^*)/(c_i + K) - b]$. As V_{cmax} increases, all else equal, A_n also increases – up to the point where $A_J = A_C$. However, if $A_J < A_C$, then A_n is given by $(J/4)\,(c_i - \Gamma^*)/(c_i + 2\Gamma^*)$. As V_{cmax} increases further, all else equal, A declines due to increasing R_d. Therefore, the value of V_{cmax} for which $A_J = A_C$ is the value that maximizes A_n.

Notes S2. Derivation of the optimal stomatal ratio χ and the sensitivity parameter ξ

The P-model (Prentice *et al.*, 2014) predicts the optimal ratio of leaf-internal to ambient CO_2 partial pressure ($\chi = c_i/c_a$) based on the hypothesis that plants minimize the combined unit costs of transpiration (E) *and* carboxylation capacity (V_{cmax}) for a given assimilation rate (A) . The optimal balance is achieved when the marginal cost of water loss equals the marginal cost of maintaining carboxylation capacity:

$$\frac{\partial(E/A)}{\partial\chi} = -\frac{b}{a}\frac{\partial(V_{cmax}/A)}{\partial\chi} \qquad (S1.1)$$

where a and b are the unit costs for water transport and carboxylation, respectively. b is assumed to be constant, and a is temperature dependent with water viscosity. Based on Fick's law ($E = 1.6g_sD$) (Fick, 1855) and the diffusion equation ($A = g_sc_a(1-\chi)$), the marginal water cost is:

$$\frac{\partial(E/A)}{\partial\chi} = \frac{1.6D}{c_a(1-\chi)^2} \qquad (S1.2)$$

Based on the FvCB model (Farquhar *et al.*, 1980) for Rubisco-limited assimilation, and applying the simplification $\Gamma^* \ll c_a$ for the derivative, the marginal carbon cost is:

$$\frac{\partial(V_{cmax}/A)}{\partial\chi} \approx -\frac{K}{c_a\chi^2} \qquad (S1.3)$$

Then the marginal costs (Eq. S1.1) can be rewritten into

$$a'\eta^*\frac{1.6D}{c_a(1-\chi)^2} = b\frac{K}{c_a\chi^2} \qquad (S1.4)$$

Solving the Eq. S1.4 and rearranging terms leads to the solution for χ

$$\frac{\chi^2}{(1-\chi)^2} = \frac{bK}{a'\eta^* 1.6D} \quad (S7)$$

We introduce the parameter $\beta = b/a'$. Taking the square root of both sides gives:

$$\frac{\chi}{1-\chi} = \sqrt{\frac{\beta K}{1.6\eta^* D}} \quad (S1.5)$$

From the solution in Eq. S1.5, we define the term on the right-hand side (excluding D)as the parameter ξ. This parameter determines the sensitivity of the optimal χ to vapour pressure deficit (D)

$$\xi = \sqrt{\frac{\beta K}{1.6\eta^*}} \quad (S1.6)$$

The optimal χ can be derived as

$$\chi = \frac{\xi}{\xi + \sqrt{D}} \quad (S1.7)$$

Without the assumption of Γ^* = 0, and following similar deduction steps, the optimal χ is

$$\chi = \frac{\Gamma^*}{c_a} + \left(1 - \frac{\Gamma^*}{c_a}\right)\frac{\xi}{\xi + \sqrt{D}} \quad (S1.8)$$

ξ is calculated as

$$\xi = \sqrt{\frac{\beta(K + \Gamma^*)}{1.6\eta^*}} \quad (S1.9)$$

In this way, we get the same format as Eqn15.

Notes S3. Derivation of V_{cmax} and J_{max}

Based on the standard FvCB model formulation (Farquhar *et al.*, 1980),

$$A_c = V_{cmax}\, m_C \quad (S2.1)$$

$$A_J = \frac{J}{4}\, m \quad (S2.2)$$

where $m_C = \frac{c_i-\Gamma^*}{c_i+K}$ and $m = \frac{c_i-\Gamma^*}{c_i+2\Gamma^*}$ are the CO_2 limitation terms for carboxylation and electron transport, respectively.

In the linear region of the light response curve (low light conditions), the electron transport rate (J) is proportional to absorbed photosynthetically active radiation I_{abs}.

$$J \approx 4\varphi_0 I_{abs} \quad (S2.3)$$

where φ_0 is the intrinsic quantum yield. Substituting Eq. S2.3 into Eq. S2.2 yields the linear form similar to a light use efficiency model:

$$A_J = \varphi_0 I_{abs} m \quad (S2.4)$$

To account for the saturation of electron transport at high light, we modify Eq. S2.4 with the non-rectangular hyperbola formulation (Smith, 1937):

$$A_J = \varphi_0 I_{abs} m \frac{1}{\sqrt{\left(1 + \left(\frac{(4\varphi_0 I_{abs})}{J_{max}}\right)^2\right)}} \quad (S2.5)$$

The optimization of J_{max} balances the marginal benefit of carbon assimilation against the marginal cost of maintaining electron transport capacity (the least cost hypothesis), which can be written as:

$$\max\left(A_J - cJ_{max}\right) \quad (S2.6)$$

where c is the unit cost of maintaining J_{max}.

Differentiating Eq. S2.6 with respect to J_{max} and setting the result to zero maximizes the profit. This implies that the marginal gain in assimilation equals the marginal cost:

$$\frac{\partial A_J}{\partial J_{max}} = c \tag{S2.7}$$

Differentiating equation S2.7 and substituting it into equation S2.5 gives:

$$c = \frac{m(\varphi_0 I_{abs})^3}{4\left[(\varphi_0 I_{abs})^2 + \left(\frac{J_{max}}{4}\right)^2\right]^3} \tag{S2.8}$$

Rearranging equation S2.8 allows us to solve for the J_{max} limitation factor (L), which modifies the effective light use efficiency:

$$L = \frac{1}{\sqrt{1 + \left(\frac{4\varphi_0 I_{abs}}{J_{max}}\right)^2}} = \sqrt{1 - \left(\frac{c^*}{m}\right)^{2/3}} \tag{S2.9}$$

where $c^* = 4c$.

Rearranging this equation to isolate J_{max}:

$$J_{max} = 4\varphi_0 I_{abs} \frac{\sqrt{1 - \left(\frac{c^*}{m}\right)^{2/3}}}{\left(\frac{c^*}{m}\right)^{1/3}} \tag{S2.10}$$

Tidy up, we get

$$J_{max} = \frac{4\phi_0 I_{abs}}{\sqrt{\frac{\mathbf{1}}{\left\{1 - \left[c^* \frac{(c_i + 2\Gamma^*)}{(c_i - \Gamma^*)}\right]^{\frac{2}{3}}\right\}}} - 1} \tag{S2.11}$$

Finally, according to the coordination hypothesis (Haxeltine & Prentice, 1996; Maire *et al.*, 2012) which assumes that A_C equals to A_J under daily time average conditions. The optimal V_{cmax} is derived as:

$$V_{cmax} = \frac{A_J}{m_C} = \varphi_0 I_{abs} \frac{m\sqrt{1 - \left(\frac{4c}{m}\right)^{2/3}}}{m_C} \qquad (S2.12)$$

Tidy up, we get

$$V_{cmax} = \varphi_0 I_{abs} \frac{c_i + K}{c_i + 2\Gamma^*} \sqrt{1 - \left(\frac{4c(c_i + 2\Gamma^*)}{c_i - \Gamma^*}\right)^{2/3}} \qquad (S2.13)$$

References:

Aubinet M, Chermanne B, Vandenhaute M, Longdoz B, Yernaux M, Laitat E. **2001**. Long term carbon dioxide exchange above a mixed forest in the Belgian Ardennes. *Agricultural and Forest Meteorology* **108**: 293–315.

Bernacchi CJ, Pimentel C, Long SP. **2003**. In vivo temperature response functions of parameters required to model RuBP-limited photosynthesis. *Plant, Cell & Environment* **26**: 1419–1430.

Bernacchi CJ, Singsaas EL, Pimentel C, Portis Jr AR, Long SP. **2001**. Improved temperature response functions for models of Rubisco-limited photosynthesis. *Plant, Cell & Environment* **24**: 253–259.

Black T. **2018**. AmeriFlux AmeriFlux CA-Oas Saskatchewan - Western Boreal, Mature Aspen.

Crous KY, Drake JE, Aspinwall MJ, Sharwood RE, Tjoelker MG, Ghannoum O. **2018**. Photosynthetic capacity and leaf nitrogen decline along a controlled climate gradient in provenances of two widely distributed Eucalyptus species. *Global Change Biology* **24**: 4626–4644.

Crous KY, Drake JE, Aspinwall MJ, Sharwood RE, Tjoelker MG, Ghannoum O. **2018**. Photosynthetic capacity and leaf nitrogen decline along a controlled climate gradient in provenances of two widely distributed Eucalyptus species. *Global Change Biology* **24**: 4626–4644.

Dunn AL, Barford CC, Wofsy SC, Goulden ML, Daube BC. **2007**. A long-term record of carbon exchange in a boreal black spruce forest: means, responses to interannual variability, and decadal trends. *Global Change Biology* **13**: 577–590.

Dushan P. Kumarathunge, Medlyn BE, Drake JE, Tjoelker MG, Aspinwall MJ, Battaglia M, Cano FJ, Carter KR, Cavaleri MA, Cernusak LA, *et al.* **2018**. ACi-TGlob_V1.0: A Global dataset of photosynthetic CO2 response curves of terrestrial plants. : 18493080 Bytes.

Farquhar GD, von Caemmerer S, Berry JA. **1980**. A biochemical model of photosynthetic CO2 assimilation in leaves of C 3 species. *Planta* **149**: 78–90.

Fick A. **1855**. V. *On liquid diffusion*. *The London, Edinburgh, and Dublin Philosophical Magazine and Journal of Science* **10**: 30–39.

Ghannoum O, Phillips NG, Sears MA, Logan BA, Lewis JD, Conroy JP, Tissue DT. **2010**. Photosynthetic responses of two eucalypts to industrial-age changes in atmospheric [CO_2] and temperature. *Plant, Cell & Environment* **33**: 1671–1681.

Gough CM, Hardiman BS, Nave LE, Bohrer G, Maurer KD, Vogel CS, Nadelhoffer KJ, Curtis PS. **2013**. Sustained carbon uptake and storage following moderate disturbance in a Great Lakes forest. *Ecological Applications* **23**: 1202–1215.

Haxeltine A, Prentice IC. **1996**. A General Model for the Light-Use Efficiency of Primary Production. *Functional Ecology* **10**: 551.

Huber ML, Perkins RA, Laesecke A, Friend DG, Sengers JV, Assael MJ, Metaxa IN, Vogel E, Mareš R, Miyagawa K. **2009**. New International Formulation for the Viscosity of H2O. *Journal of Physical and Chemical Reference Data* **38**: 101–125.

Knohl A, Schulze E-D, Kolle O, Buchmann N. **2003**. Large carbon uptake by an unmanaged 250-year-old deciduous forest in Central Germany. *Agricultural and Forest Meteorology* **118**: 151–167.

Kurbatova J, Li C, Varlagin A, Xiao X, Vygodskaya N. 2008. Modeling carbon dynamics in two adjacent spruce forests with different soil conditions in Russia. *Biogeosciences* **5**: 969–980.
Leuning R, Cleugh HA, Zegelin SJ, Hughes D. 2005. Carbon and water fluxes over a temperate Eucalyptus forest and a tropical wet/dry savanna in Australia: measurements and comparison with MODIS remote sensing estimates. *Agricultural and Forest Meteorology* **129**: 151–173.
Maire V, Martre P, Kattge J, Gastal F, Esser G, Fontaine S, Soussana J-F. 2012. The Coordination of Leaf Photosynthesis Links C and N Fluxes in C3 Plant Species (B Bond-Lamberty, Ed.). *PLoS ONE* **7**: e38345.
Merbold L, Eugster W, Stieger J, Zahniser M, Nelson D, Buchmann N. 2014. Greenhouse gas budget (CO_2 , CH_4 and N_2 O) of intensively managed grassland following restoration. *Global Change Biology* **20**: 1913–1928.
Prentice IC, Dong N, Gleason SM, Maire V, Wright IJ. 2014. Balancing the costs of carbon gain and water transport: testing a new theoretical framework for plant functional ecology (J Penuelas, Ed.). *Ecology Letters* **17**: 82–91.
Prescher A-K, Grünwald T, Bernhofer C. 2010. Land use regulates carbon budgets in eastern Germany: From NEE to NBP. *Agricultural and Forest Meteorology* **150**: 1016–1025.
Smith EL. 1937. THE INFLUENCE OF LIGHT AND CARBON DIOXIDE ON PHOTOSYNTHESIS. *Journal of General Physiology* **20**: 807–830.
Smith NG, Dukes JS. 2017. Short-term acclimation to warmer temperatures accelerates leaf carbon exchange processes across plant types. *Global Change Biology* **23**: 4840–4853.
Suni T, Rinne J, Reissell A, Keronen P, Rannik U, Dal Maso M, Kulmala M, Vesala T, Altimir N. 2003. Long-term measurements of surface fluxes above a Scots pine forest in Hyytiaelae, southern Finland, 1996-2001. *Boreal Environment Research* **8**.
Stocker BD, Wang H, Smith NG, Harrison SP, Keenan TF, Sandoval D, Davis T, Prentice IC. 2020. P-model v1.0: an optimality-based light use efficiency model for simulating ecosystem gross primary production. *Geoscientific Model Development* **13**: 1545–1581.
Urbanski S, Barford C, Wofsy S, Kucharik C, Pyle E, Budney J, McKain K, Fitzjarrald D, Czikowsky M, Munger JW. 2007. Factors controlling CO_2 exchange on timescales from hourly to decadal at Harvard Forest. *Journal of Geophysical Research: Biogeosciences* **112**: 2006JG000293.
Wang H, Prentice IC, Keenan TF, Davis TW, Wright IJ, Cornwell WK, Evans BJ, Peng C. 2017. Towards a universal model for carbon dioxide uptake by plants. *Nature plants* **3**: 734–741.
